# Injectable hydrogels based on mussel-inspired nanocomposite microspheres for non-compressible intra-abdominal hemorrhage control

**DOI:** 10.7150/thno.118901

**Published:** 2025-07-28

**Authors:** Tao Liu, Peng Ma, Fengya Jing, Yinghua Tao, Jinfang Lu, Dandan Wei, Liuxin Yang, Feiling Feng, Yonghua Li, Hongbin Yuan, Tianzhu Zhang

**Affiliations:** 1 State Key Laboratory of Digital Medical Engineering, School of Biological Science and Medical Engineering, Southeast University, Nanjing 210096, China; 2 Department of Anesthesiology, Changzheng Hospital, Second Affiliated Hospital, Naval Medical University, Shanghai 200003, China; 3 Department of Biliary Tract Surgery I, Third Affiliated Hospital, Naval Medical University, Shanghai 200438, China; 4 Advanced Ocean Institute of Southeast University, Nantong 226010, China

**Keywords:** cryogel microspheres, mussel-inspired strategy, nanocomposite, injectable hydrogels, rapid hemostasis

## Abstract

**Background:** The development of hemostatic materials for non-compressible intra-abdominal hemorrhage in complex pre-hospital emergency settings remains a formidable challenge.

**Methods:** A novel injectable hydrogel based on mussel-inspired nanocomposite microspheres was designed. The biocompatible hydrogel was formed by hydrating gelatin methacryloyl (GelMA) cryogel microspheres—reinforced with polydopamine (PDA)-intercalated nanoclay—with sterile saline, offering the dual benefits of convenient storage of microspheres and precise delivery to deep bleeding points via injection.

**Results:** The cryogel microspheres, featuring rapid water and blood absorption within 1 second, exhibited outstanding procoagulant capabilities in both *in vitro* and *in vivo* experiments, showing potential as hemostatic agents for open wounds. Notably, the on-demand formulated hydrogel effectively controlled severe bleeding within 2 minutes post-injection in rat liver volumetric defect and partial resection models, demonstrating ​significantly enhanced applicability compared to ​bulk hemostatic agents for irregular wounds. Additionally, ultrasound-guided application in a porcine liver ​rupture model confirmed that the hydrogel rapidly filled and sealed deep wounds, interacted with blood components to form stable, large clots adhering to the wound surface, and thus established durable hemostasis.

**Conclusion:** This study presents a promising injectable hemostatic material for pre-hospital emergency hemorrhage control.

## Introduction

It is estimated that uncontrolled post-traumatic hemorrhage is responsible for the deaths of approximately 1.5 million individuals globally each year 1. Severe trauma frequently results in the “Trauma Triad of Death”, including hypothermia, acidosis, and coagulopathy 2, 3, which underscores the critical importance of achieving hemorrhage control within the “golden hour” of post-trauma 4. In both civilian and battlefield settings, over one-third of hemorrhagic deaths are caused by non-compressible intra-abdominal hemorrhage (NCIAH) 5. At present, NCIAH can only be definitively treated by surgical intervention 6. There are limited strategies that can be employed in the pre-hospital setting to prolong survival and enabling patients to undergo surgery.

Hemostatic materials play an important role in the management of bleeding in pre-hospital emergency care. However, traditional materials like tourniquets, gauze, and sponges struggle to access intra-abdominal deep wounds and occult bleeding sites, therefore failing to achieve rapid and effective hemostasis 7-9. The treatment of intracavitary hemorrhage necessitates the utilization of minimally invasive techniques. Injectable hemostatic materials have garnered significant attention in recent years due to their ability to treat invisible or inaccessible wounds 10. These fluid forms of hemostatic agents can rapidly establish complete contact with irregularly-shaped hemorrhagic lesions, rendering them particularly suitable for NCIAH management 11. Nevertheless, existing injectable hemostats are predominantly ​formulated as prefabricated solutions or sol preparations. The stringent storage and transportation requirements of these liquid formulations pose significant challenges in resource-limited scenarios such as naval warfare. This necessitates the development of novel injectable hemostatic agents to convenient, rapid hemorrhage control in pre-hospital settings and prolong patients' pre-surgical survival time.

Gelatin, a product derived from partial hydrolysis of natural polymeric collagen, has been widely used in hemostatic applications due to its favorable biocompatibility, biodegradability and cell-adhesive properties 12. Several absorbable gelatin-based sponges are currently marketed (e.g., Gelfoam, Pfizer) and approved for hemostasis in surgical wounds 13. However, the rapid degradation and poor mechanical properties of gelatin at physiological temperatures significantly limit its biomedical applications 14. Gelatin methacryloyl (GelMA), a gelatin derivative, forms covalently crosslinked hydrogels with tunable mechanical properties via UV-initiated radical polymerization in the presence of photoinitiators 15. This property has led to the widespread use of GelMA in constructing injectable hydrogel materials. Despite combining the advantages of both natural and synthetic materials, GelMA hydrogels do not exhibit significant hemostatic properties. Several studies have reported the combination of Laponite® (Lap), a nanosilicate from the smectite family, with GelMA to develop novel hemostatic materials 16-19. Lap, a nanoplatelet with a negatively charged surface and positively charged edge (20-30 nm in diameter and about 1 nm in thickness), induces blood coagulation by concentrating coagulation factors and degrades in the body into non-toxic, bioabsorbable minerals 20-22. The alkaline nature of the aqueous dispersion of such nanoclay enables *in situ* oxidation of dopamine (DA) embedded within the confined nanospace between the two-dimensional layers of Lap, resulting in polydopamine (PDA)-intercalated nanosheets with free catechol groups 23, 24. This mussel-inspired strategy could further enhance the mechanical and adhesive properties of injectable hydrogel materials.

**Scheme 1:**
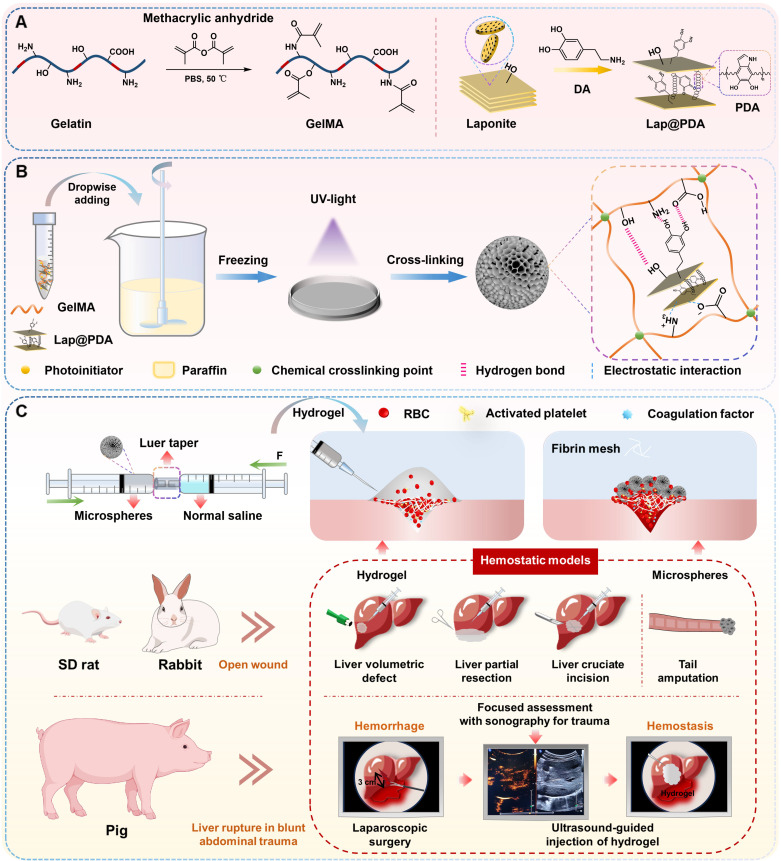
Schematic diagram of the preparation and hemostatic application of injectable microsphere-based hydrogels. (A) Synthesis of GelMA and Lap@PDA. (B) Preparation of GM-Lap@PDA cryogel microspheres. Lap@PDA serves as the core physical crosslinking hub. PDA provides catechol and amino groups for hydrogen bonding with GelMA chains, while electrostatic interactions and hydrogen bonding exist between Laponite and GelMA. GelMA photopolymerization forms the covalent network. These synergistic physical and chemical interactions ensure stable microsphere formation. (C) Preparation and application of GM-Lap@PDA hydrogels. Several representative hemorrhage models were used to investigate the hemostatic potential of microspheres/hydrogels on open or invisible wounds.

Here, we developed a novel injectable microsphere-based hydrogel system for emergency hemostasis in NCIAH (Scheme 1). This system offers ​distinct structural and functional advantages over conventional injectable hemostats: (1) The core is based on dried porous cryogel microspheres​ composed of mussel-inspired Lap@PDA and GelMA. These microspheres, designated as GM-Lap, GM-Lap@PDA_I_ and GM-Lap@PDA_II_ corresponding to increasing amounts of DA, were prepared via water-in-oil (W/O) emulsification, gradient freezing, and UV cross-linking (Scheme 1A and B). They possess remarkable properties in terms of portability, ease of sub-packaging, and long-term storability, ideal for use in resource-limited or battlefield environments. (2) The cryogel microspheres absorb water and blood within 1 second and demonstrate remarkable procoagulant activity in both *in vitro* and *in vivo* settings, making them effective for open wound management. (3) Critically, these microspheres undergo controllable saline-mediated hydration, transforming within seconds into an injectable hydrogel. ​This on-demand process enables rapid deployment in pre-hospital or field settings, with immediate delivery capability via a 20 G needle into deep, irregular wounds (Scheme 1C). (4) The resulting hydrogel can be injected under ultrasound guidance near the bleeding site within the abdominal cavity to rapidly fill and seal deep wounds, interact with blood components to form a stable clot adhering to the wound bed, and thereby achieve durable hemostasis. We systematically characterized DA content effects on injectability, mechanics, adhesion, and biocompatibility of hydrogels, while the emergency hemostatic efficacy was rigorously validated across multiple representative NCIAH models including:​​ (1) rat liver volumetric defect models; (2) rat liver partial resection models; (3) rat/rabbit liver cruciate incision models, and (4) porcine liver rupture models simulating blunt abdominal trauma. The final results demonstrated that the mussel-inspired nanocomposite microsphere-based hydrogel is biologically safe and degradable, ​rendering it suitable for complex and variable pre-hospital emergency scenarios.

## Results and Discussion

### Preparation and characterization of GM-Lap@PDA cryogel microspheres

The proposed fabrication process of GM-Lap@PDA cryogel microspheres was illustrated in Scheme 1A and B. GelMA was synthesized via methacryloyl group modification of primary amines and hydroxyl groups on gelatin (Scheme 1A and Figure S1A). And the RGD motifs in gelatin remained non-reactive toward MA, thereby preserving the intrinsic cell adhesion properties of GelMA 25. The successful synthesis of GelMA was confirmed by ^1^H-NMR spectrum, revealing two characteristic peaks at δ = 5.65 ppm and δ = 5.41 ppm corresponding to the acrylic protons of methacryloyl groups (Figure S1B). The DM of GelMA was calculated to be about 55% based on the ^1^H-NMR.

The synthesis of PDA-intercalated nanoclay (Lap@PDA) was performed according to previously reported protocols 23, 24. Upon dispersing DA in an alkaline nanoclay (Lap) suspension, the mixture transitioned from colorless to dark brown, indicating *in situ* oxidative self-polymerization of DA into PDA (Figure S2). The lyophilized Lap@PDA composites were subsequently characterized in terms of chemical structure and microscopic morphology. Comparing the FT-IR spectrum of Lap (Figure S3A), a new characteristic peak emerged at 1517 cm^-1^ in the Lap@PDA spectrum, assignable to the N-H scissoring vibration of PDA 26, 27. The XRD patterns of Lap, Lap@PDA, GM-Lap, and GM-Lap@PDA were presented in Figure S3B. The characteristic diffraction peak (2θ = 6.1°) of (001) crystalline plane of pure nanoclay (Lap) corresponded to a d-spacing of 1.4 nm. For Lap@PDA, the characteristic peak was left-shifted to 2θ = 5.9° with the d _(001)_ spacing of about 1.5 nm. The slight increase in the d-spacing confirmed that PDA intercalated into the nanoclay interlayer. Moreover, the XRD patterns of GM-Lap and GM-Lap@PDA were almost featureless. This could be attributed to complete exfoliation of the nanoplatelets during polymer preparation and their uniform dispersion in the polymer-Lap/Lap@PDA hybrids 23. Further, the nanolayered architectures of Lap and Lap@PDA were observed by FE-SEM (Figures S4 and S5). Lap@PDA exhibited well-defined lamellar morphology, whereas Lap nanoplatelets displayed irregularly aggregation. EDS elemental mapping confirmed uniform distribution of C and N within Lap@PDA alongside the characteristic O, Si, and Mg signatures inherited from the nanoclay matrix (Lap).

**Figure 1:**
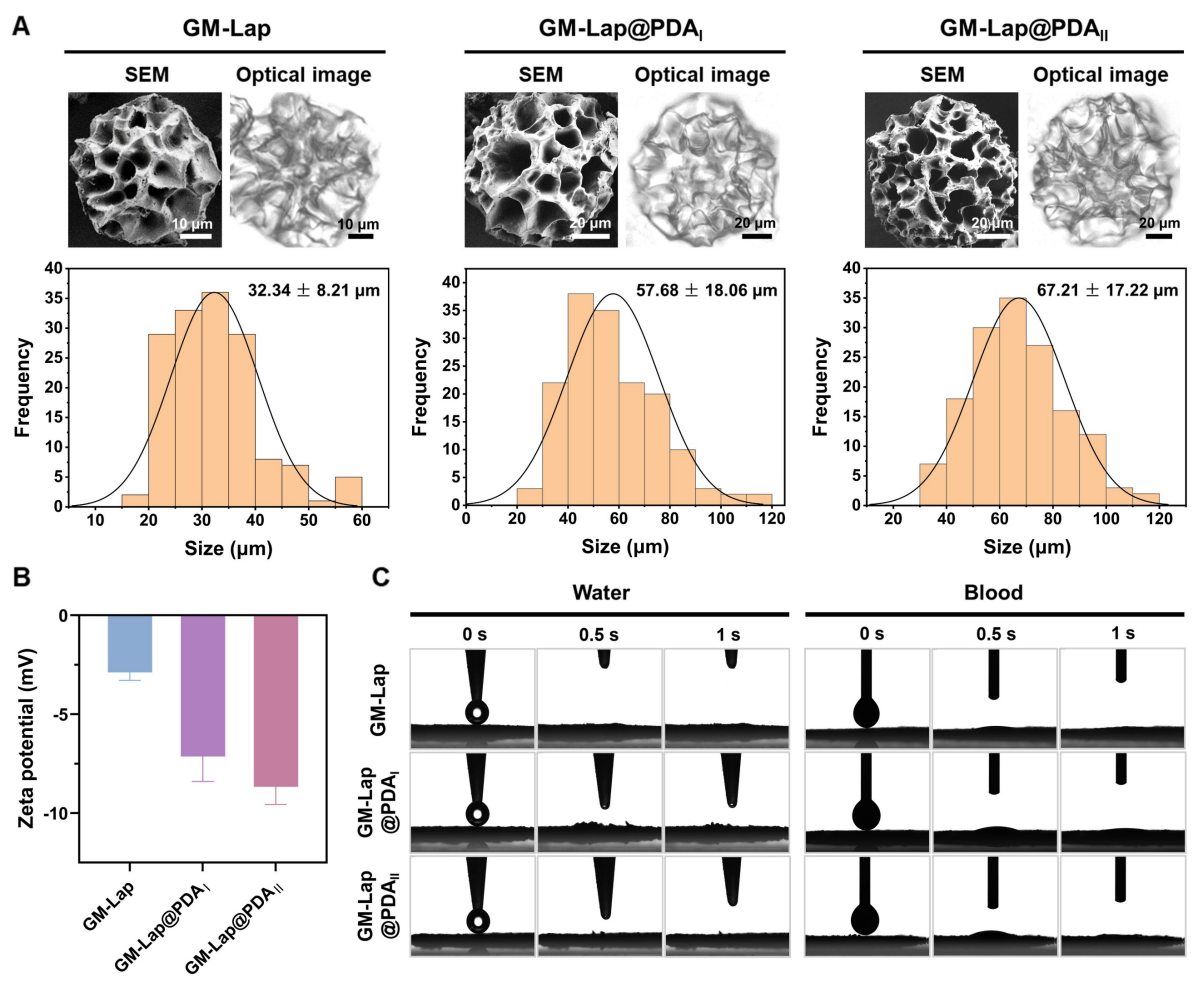
Physical characterization of GM-Lap@PDA cryogel microspheres. (A) The morphology images and particle size distribution of microspheres. (B) Zeta potential of microspheres (n = 3). (C) Absorption of water or blood by microspheres in 1 s.

GM-Lap@PDA cryogel microspheres were fabricated through a three-step protocol involving W/O emulsification, gradient freezing, and UV cross-linking (Scheme 1B) 28, 29. The emulsion was first cooled to 4 °C to induce physical gelation. Subsequently, it was frozen at -20 °C to allow controlled ice crystal formation, followed by rapid quenching in liquid nitrogen (-196 °C) to halt further crystal growth and complete the freezing process. The frozen solvent crystals acted as porogens, facilitating the formation of open and interconnected macroporous structures in the microspheres. The SEM and optical images showed that all three microspheres exhibited similar spherical morphology and macroporous structure (Figure 1A). Furthermore, the average diameters of the microspheres increased with higher DA content, but all remained below 100 μm with a narrow particle size distribution. This size increase may be related to stronger non-covalent interactions within the aqueous phase caused by Lap@PDA. The chemical composition of microspheres was verified by FT-IR (Figure S6A). The characteristic peaks of GelMA were observed in the spectra of all three samples, including the stretching vibrational peak of -C=O at 1655 cm^-1^ (amide I) and the bending vibrational peak of N-H at 1534 cm^-1^ (amide II) 30. Additionally, the peaks at 443 cm⁻¹ and 1000 cm⁻¹ in the spectra of the composite samples corresponded to the asymmetric stretching and symmetric bending vibrations of Si-O-Si bonds in Lap, respectively 27. The zeta potential of the composite cryogel microspheres was measured (Figure 1B). All three microspheres exhibited negative surface charges, with GM-lap@PDA_II_ showing a zeta potential nearly three times higher in magnitude than that of GM-Lap. This difference could be attributed to the inherent negative charge of Lap and free catechol groups introduced by PDA in Lap@PDA 31, 32. It has been reported that negative charged moieties in biomaterials induce blood coagulation 18, 33.

### Absorption properties of microspheres

The absorption capacity of GM-Lap@PDA cryogel microspheres was assessed by monitoring changes in dynamic water/blood contact angle over 1 s (Figure 1C). Evidently, all microspheres were able to completely absorb the droplets within 1 s (Movies S1 and S2). This was due to the presence of hydrophilic groups (e.g., phenolic hydroxyl, hydroxyl and amino groups) on the microspheres' surface, which, combined with the macroporous structure, synergistically endowed the microspheres with superhydrophilic/hemophilic properties. When used as a powder hemostatic agent, the excellent liquid absorption capability of microspheres facilitates blood concentration, hemocyte aggregation and retention of coagulation components 34. The swelling properties of the cryogel microspheres were further evaluated. After immersion in saline, the three microspheres rapidly absorbed water and swelled, reaching swelling rates of 544%, 432%, and 389%, respectively, within 10 minutes (Figure S6B). The incorporation of Lap@PDA enhanced the cross-linking density within the cryogel microspheres, thereby restricting their further swelling.

### Preparation and characterization of injectable microsphere-based hydrogels

The series of characterizations demonstrated that GM-Lap@PDA cryogel microspheres possess rapid liquid absorption capacity and procoagulant potential. To advance the treatment of non-compressible intra-abdominal hemorrhage, we have developed an injectable microsphere-based hydrogel system designed to achieve minimally invasive, precise delivery of active hemostatic agents to the deep bleeding sites.

**Figure 2:**
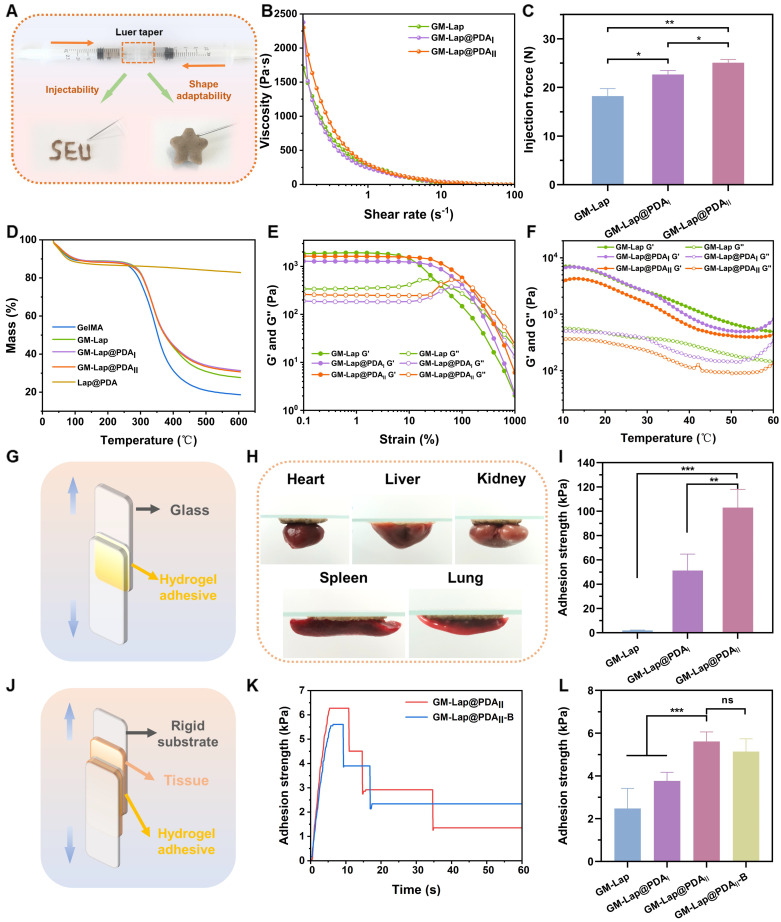
Physicochemical characterization and adhesion properties of injectable microsphere-based hydrogels. (A) Schematic diagram of the device for the preparation of injectable hydrogels. (B) Shear thinning property test. (C) Injection force of the hydrogel through a 20 G needle at a rate of 2 mL min^-1^ (n = 3). (D) The thermogravimetric analysis (TGA) curves. (E) Oscillatory strain scan at a constant angular frequency of 10 rad s^-1^. (F) Temperature stability test. (G) Schematic diagram of the device for lap-shear adhesion test (glass slide). (H) Adhesion of GM-Lap@PDA_II_ hydrogel to the surface of different organ tissues. (I) Adhesion strength of GM-Lap@PDA hydrogels to glass slides (n = 4). (J) Schematic diagram of the device for lap-shear adhesion test (porcine skin). (K) The adhesion strength-time curve of GM-Lap@PDA_II_ hydrogel to porcine skin in different environments. (L) Adhesion strength of GM-Lap@PDA hydrogels to porcine skin (n = 4). **p < 0.01, ***p < 0.001, and ns indicates no significant difference between groups.

Dried microspheres and saline were loaded into two syringes connected via a Luer taper at specific mass-to-volume ratios. The syringes were rapidly pushed back and forth until homogeneous mixing was achieved, at which point the pre-gel could be injected through a 20 G needle (within 30 seconds total mixing/injection time, Movie S3) and quickly formed *in situ* into a hydrogel conforming to the mold geometry (Figure 2A), demonstrating that the GM-Lap@PDA hydrogel was shape-adaptive. As shown in Figure 2B, the three hydrogels all showed shear-thinning characteristics, wherein viscosity declines with increasing shear rate, a key property for injectability. This shear-thinning and shape-adaptive hydrogel can be injected directly into the intracavitary injury site, fully contacting and accurately filling a variety of irregular wounds and gaps 35. The shear-thinning behavior originates from non-covalent interactions within the hydrogel. These include dynamic formation/breakage of self-assembled structure of Lap nanoplatelets driven by electrostatic interactions, as well as reversible interactions between PDA molecules such as π-π stacking and hydrogen bonding 36, 37. The suitable injectability of hydrogels contributes to improved handling efficiency and ​reduced medical costs 38. For this, the injection force required to extrude the hydrogel through a 20 G needle at a rate of 2 mL min^-1^ was measured (Figure 2C). GM-Lap@PDA_II_ exhibited the highest injection force among the tested hydrogels. This result may be attributed to the catechol group-induced enhancement of hydrogel's cross-linking density, which necessitates elevated shear stress for dynamic network disruption 39. Nevertheless, the injection force of GM-Lap@PDA_II_ remained within the range that could be applied by hand 40. Subsequently, the thermal stability of the hydrogels was characterized by thermogravimetric analysis. As shown in Figure 2D, all four gels showed similar thermal weight loss behavior under N_2_ atmosphere. The mass loss of the samples ranged from 70% to 82% at the final temperature of 600 °C, with the order of mass loss being GelMA > GM-Lap > GM-Lap@PDA_I_ similar to GM-Lap@PDA_II_. These results suggested that the incorporation of Lap@PDA strengthened the non-covalent interactions within the hydrogel, thereby enhancing its thermal stability. In addition, since Lap is a silicate inorganic material that does not decompose easily at high temperatures, the final residual mass of GM-lap@PDA hydrogels was higher than that of GelMA.

To evaluate the mechanical performance, rheological experiments were conducted on the hydrogels. Oscillatory strain scans were performed on the hydrogels at a constant angular frequency (Figure 2E). The intersection of the storage modulus (G′) and loss modulus (G″) curves represents the gel-sol transition point 41. When the strain applied by the rheometer exceeds the critical strain of the hydrogel, G′ becomes smaller than G″ and decrease sharply, indicating that the gel structure collapses. The higher the critical strain, the stronger the deformation resistance of the gel 42. As evidenced in Figure 2E, the critical strain of the GM-Lap@PDA hydrogels progressively increased with higher DA content. This result demonstrated that Lap@PDA, acting as a functional nanofiller incorporated into the GelMA network, enhances the hydrogel's cohesion and structural stability. The contrast between ​the loosely connected structure composed of fragmented microspheres observed in the GM-Lap hydrogel system and the highly interconnected porous structure presented by the GM-Lap@PDA_II_ hydrogel further demonstrated that the presence of PDA significantly affects the internal cross-linking within the hydrogel (Figure S7). Mechanistically, hydration initiates gelation by ​rapidly swelling the microspheres and inducing tight interfacial contact. This proximity facilitates crosslinking between the photocrosslinked GelMA network and Lap via hydrogen bonding and electrostatic interactions. Concurrently, PDA mediates interfacial fusion through multiple mechanisms: (i) hydrogen bonding between catechol groups and amino/carboxyl/ hydroxyl moieties on GelMA or hydroxyl groups on Lap, (ii) chelation of ​Lap-sourced​ metal ions (e.g., Mg²⁺)​ by​ catechol groups, and (iii) π-π stacking between aromatic structures 23. Collectively, these synergistic mechanisms effectively enhanced the ​overall mechanical performance​ of the GM-Lap@PDA hydrogel.

The temperature stability of the hydrogels was tested over a range of common environmental and physiological temperatures (10-60 ℃) to which they may be exposed during *in vitro* or *in vivo* application (Figure 2F). The storage modulus of all three gels decreased to varying degrees with increasing temperature, which is related to the temperature sensitivity of GelMA 43.

### Lap shear adhesion properties of hydrogels

Hydrogel adhesives used for hemostasis of deep incompressible wounds, particularly in intra-abdominal organs, should rapidly form a gel *in situ* and seal the bleeding site after injection 44. First, glass slides were selected as the adhesion substrate to investigate the effect of DA content in GM-Lap@PDA hydrogel on adhesion strength through lap-shear experiments (Figure 2G). As shown in Figure 2I, the adhesion strength of GM-Lap@PDA_II_ reached 103 kPa, approximately 50 times higher than that of GM-Lap. This enhanced adhesion strength can be attributed to both the substrate material and the catechol groups. Glass slides, being low-surface-energy substrates with hydrophobic surfaces, facilitate the removal of water molecules from the adhesion interface, thereby promoting stronger physical interactions between free catechol groups and the substrate surface 45. Subsequently, the hydrogel's adhesion to various biological tissues was examined (Figure 2H and J). GM-Lap@PDA_II_ exhibited good adhesion to multiple organ surfaces (heart, liver, spleen, lungs, and kidneys) (Figure 2H). From Figure 2L, the highest lap shear adhesion strength of GM-Lap@PDA_II_ to fresh porcine skin was 5.6 kPa. Crucially, the presence of blood on the porcine skin surface (GM-Lap@PDA_II_-B versus GM-Lap@PDA_II_) did not significantly compromise the hydrogel's adhesive efficacy (Figure 2K and L). The adhesion mechanism of GM-Lap@PDA hydrogels can be summarized as follows: primarily, the coexistence of abundant catechol, amine, and imine groups in Lap@PDA contributes to the hydrogel's ​superior adhesion performance toward both inorganic/organic matrices and biological tissue surfaces 46-48; and secondly, the presence of multiple structural domains within GelMA that interact with cell-surface receptors and extracellular matrix proteins enhances its tissue-adhesive properties 49.

Comparative analysis of Figure 2I and L revealed remarkably reduced hydrogel adhesion strength to porcine skin versus glass substrates, likely attributing to the presence of both residual surface grease on biological tissue and potential interfacial hydrated layer. It should be emphasized that the uniqueness of the injectable microsphere-based hydrogel developed in this study lies in its on-demand formation through immediate hydration of microspheres with saline, achieving gelation via a surface hydration-induced contact-enhanced effect between microspheres. While this dynamic crosslinking process endows the material with favorable injectable properties, subsequent water absorption at the adhesive interface may disrupt the non-covalent interactions (e.g., hydrogen bonds) within or between molecules, thereby compromising the gel's cohesion, which directly accounts for the diminished adhesive strength 50. Notably, comparative analysis with the reported adhesive strength (0.5-7.2 kPa) of commercially available fibrin glues (Figure S8) indicates GM-Lap@PDA_II_ hydrogel achieves a certain level of clinical acceptability in tissue adhesion, while still exhibiting significant potential for improvement to address complex high-pressure bleeding scenarios.

### *In vitro* blood-clotting performance

**Figure 3:**
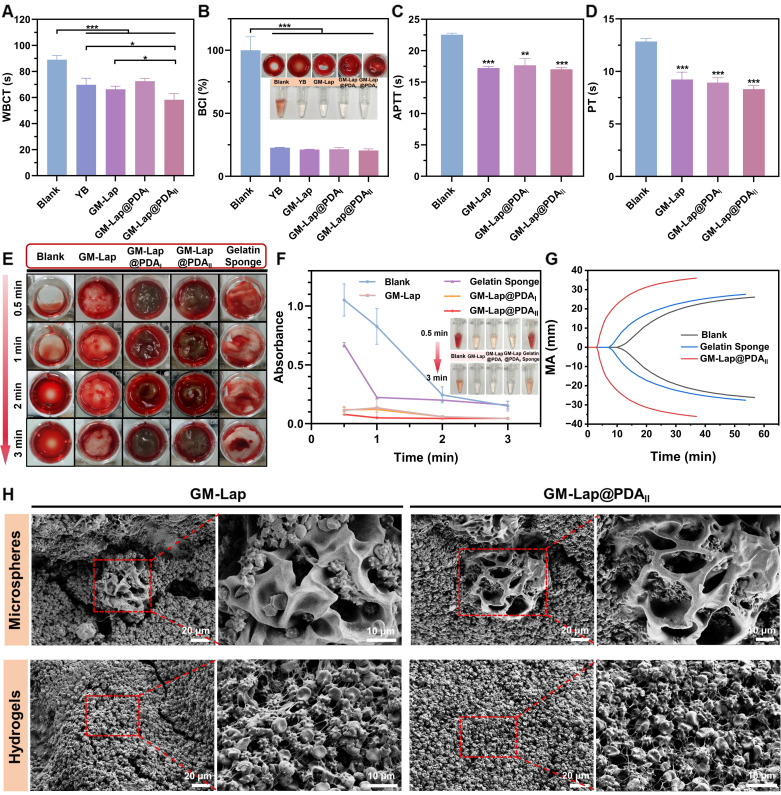
*In vitro* blood-clotting performance. (A) The whole blood clotting time (WBCT) of the blank group, GM-Lap@PDA cryogel microspheres and Yunnan Baiyao (YB) power (n = 4). (B) Blood coagulation index (BCI) of the blank group, GM-Lap@PDA cryogel microspheres and YB power at 3 min (n = 3) (*p < 0.05 and ***p < 0.001). (C, D) APTT (Activated partial thromboplastin time) and PT (prothrombin time) of the blank group and GM-Lap@PDA cryogel microspheres (n = 3) (**p < 0.01 and ***p < 0.001 vs the blank group). (E) *In vitro* coagulation status of the blank group, GM-Lap@PDA hydrogels and gelatin sponge after removal of supernatant at different time points. (F) The supernatant absorbance values of the blank group, GM-Lap@PDA hydrogels and gelatin sponge after coagulation at 0.5, 1, 2, and 3 minutes (n = 3). (G) The thromboelastogram (TEG) of the blank group, GM-Lap@PDA_II_ hydrogel and gelatin sponge (n = 3). (H) SEM images of blood cell adhesion on the surface of GM-Lap@PDA cryogel microspheres and hydrogels.

The microspheres were first subjected to *in vitro* coagulation tests with the commercial hemostatic agent YB powder serving as the positive control. The whole blood clotting time (WBCT) of each group was measured (Figure 3A), which can visually reflect the procoagulant properties 51. The WBCT of each sample group was shortened to varying degrees compared to the blank group (89 ± 2.7 s). The GM-Lap@PDA_II_ group demonstrated the shortest clotting time (58 ± 4.1 s), which was statistically significantly lower than that of YB powder (70 ± 4.3 s, *p* < 0.05). The blood coagulation index (BCI) of each group was further determined after 3 min of clotting (Figure 3B). An increased absorbance value of the hemoglobin solution corresponds to a slower coagulation rate of the sample 52. Quantitative analysis revealed remarkably reduced BCI values in all experimental groups compared to the blank control group. Notably, microspheres incorporating Lap@PDA exhibited distinct gel-like viscous substance formation upon blood contact.

The whole blood clotting kinetics test was conducted on GM-Lap@PDA hydrogels using a commercial absorbable gelatin sponge as the positive control. The procoagulant ability of each group was evaluated by continuously observing the coagulation state of whole blood in contact with the material and detecting the absorbance values of the hemoglobin supernatant at different time points (Figure 3E and F). From Figure 3E, the hydrogels undergone swelling during co-incubation with whole blood and the blood was encapsulated inside and on the surface of the gel, allowing the formation of hydrogel/clot complexes in a short period of time (30 s). Correspondingly, the supernatant absorbance values across all hydrogels at each time point were much lower than those of the blank control group and the gelatin sponge group, indicating that the GM-Lap@PDA gels could effectively induce blood coagulation (Figure 3F). In addition to this, the procoagulant capacity of GM-Lap@PDA_II_ hydrogel and gelatin sponge was further assessed by monitoring the viscoelastic changes in whole blood coagulation upon contact with the samples (Figure 3G). Thromboelastography provides several key evaluation parameters: Reaction time (R value), Clot formation time (K value), Angle (A), Maximum amplitude (MA value), and Coagulation composite index (CI value) 53. R value represents the time of sequential activation of coagulation factors during the initiation of the coagulation process (Figure S9A). K value and angle A are closely related to each other, both reflecting the rate of clot formation (Figure S9B and C). MA value indicates the maximal strength or hardness of the formed clots (Figure S9D). CI value, derived from the combination of R value, K value, angle A and MA, reflects the comprehensive coagulation state: CI > 3 indicates hypercoagulation and CI < -3 indicates hypocoagulation (Figure S9E). The hydrogel group exhibited the best overall coagulation (CI: 1.9 ± 1.0; R: 3.7 ± 0.7 min), with significantly higher clotting factors activity, compared to the blank group (CI: -10.7 ± 0.7; R: 13.2 ± 1.3 min) and gelatin sponge group (CI: -6.1 ± 0.4; R: 8.5 ± 0.3 min). Collectively, these results sufficiently confirmed the excellent *in vitro* coagulation performance of the GM-Lap@PDA_II_ hydrogel.

Active hemostatic materials positively influence the primary or secondary coagulation process 13. To investigate the potential coagulation mechanisms of GM-Lap@PDA microspheres and hydrogels, APTT/PT (activated partial thromboplastin time/prothrombin time) assays and blood cell adhesion experiments were conducted. APTT and PT are widely recognized as sensitive screening indicators for the intrinsic and extrinsic coagulation pathways, respectively. As shown in Figure 3C, the APTT values of GM-Lap@PDA microspheres were all significantly lower than those of the blank group (PPP without sample contact). Based on reported studies 33, 54, this result is strongly attributed to the negatively charged surface of the microspheres, conferred by the presence of nanosilicate (Lap) and catechol groups. Furthermore, the PT values of the microsphere group were markedly reduced compared to the blank group (Figure 3D). These findings indicated that GM-Lap@PDA microspheres can activate both intrinsic and extrinsic coagulation pathways to accelerate coagulation.

Next, scanning electron microscopy (SEM) was employed to visualize the state of blood clots on the surface of microspheres and hydrogels. Numerous blood cells were observed adhering to both surfaces and interiors of GM-Lap and GM-Lap@PDA_II_ cryogel microspheres (Figure 3H), attributable to their distinctive macroporous structures that rapidly absorb blood moisture while concentrating active ingredients (e.g., erythrocytes and platelets). After trapping blood cells and coagulation factors, the procoagulant components (Lap@PDA) further activated the coagulation cascade. This synergistic interaction between structural and chemical effects elucidates the coagulation mechanism of GM-Lap@PDA cryogel microspheres. The procoagulant ability of GM-Lap@PDA continued after the microspheres were prepared into gels. Aggregated erythrocytes and activated platelets with filamentous pseudopods were clearly observed to be intertwined, forming a dense blood cell adhesion layer on the hydrogel surface (Figure 3H). Higher-resolution SEM analysis further corroborated platelet activation across all materials, revealing adherent platelets exhibiting characteristic pseudopodia extensions (Figure S10). Activated platelets release factors that trigger adjacent platelet adhesion/aggregation and initiate coagulation cascades, playing a crucial role in primary hemostasis and thrombus formation 55. This prominent adhesion of blood components is likely due to Lap-induced enhanced protein adhesion on nanocomposite surfaces and the PDA-mediated adhesion/activation of blood cells through catechol moieties interactions with reactive residues of membrane proteins and polysaccharides via covalent/non-covalent bonding 56-58.

### *In vitro* and *in vivo* biocompatibility

Superior biocompatibility is of great benefit to hemostatic materials in clinical applications 59. The hemolysis rate is an important indicator for evaluating the hemocompatibility of the samples. *In vitro* hemolysis rate experiments were performed by co-incubating erythrocytes with hydrogels, and the results were shown in Figure S11. The hemolysis rate for all samples was less than 5%, which was within the safety limits for biological materials 60. This demonstrated that GM-Lap@PDA hydrogel used for hemostasis experiments was essentially immune to rupture of erythrocytes upon direct contact with them.

**Figure 4:**
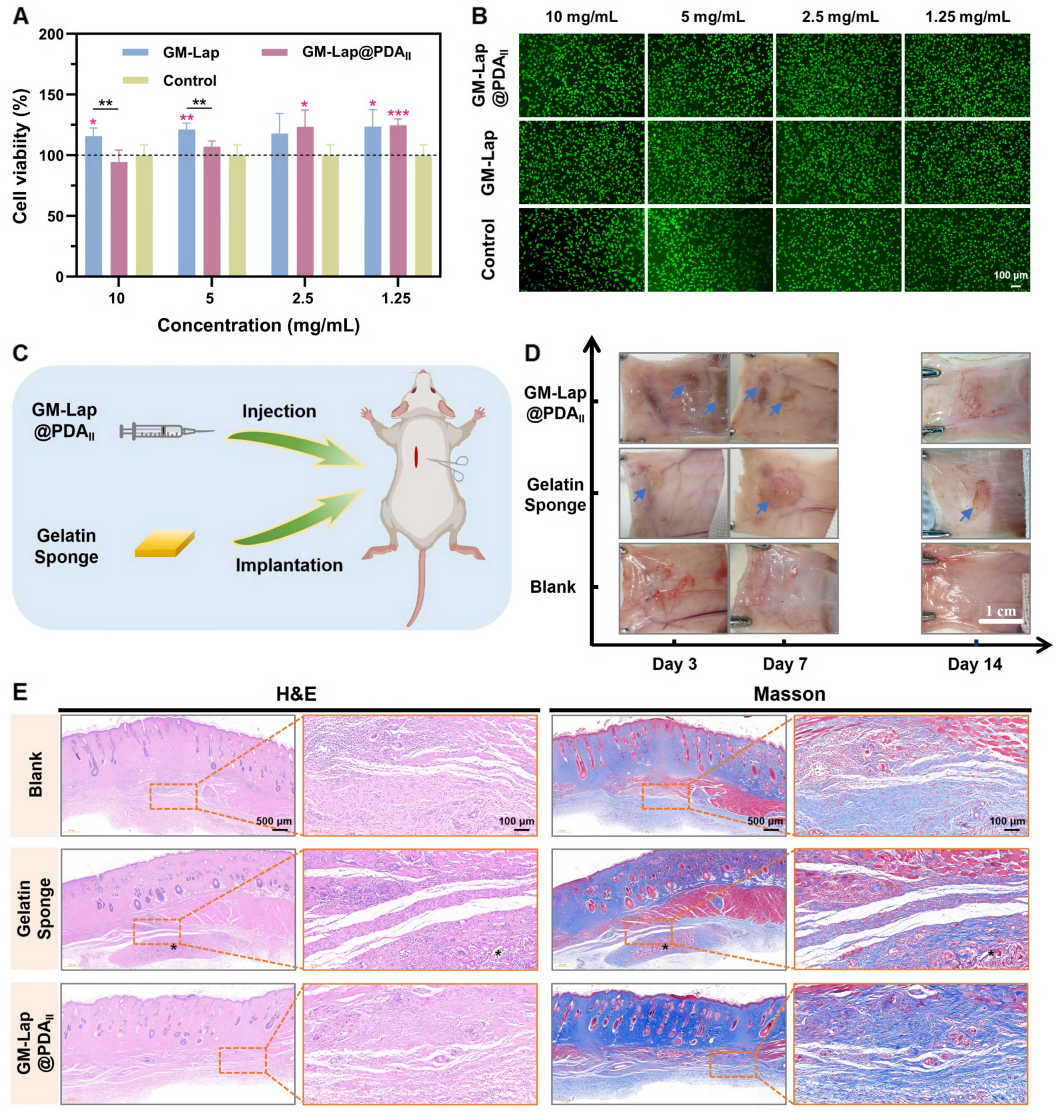
*In vitro* and *in vivo* biocompatibility. (A) Relative cell viability of L929 fibroblasts co-cultured with GM-Lap@PDA microspheres extracts for 24 h (the red asterisks represent the comparison of the experimental group with the control group at the same concentration, n = 5, *p < 0.05, **p < 0.01, and ***p < 0.001). (B) Live/dead cell fluorescence images (n = 4). (C) Schematic diagram of the subcutaneous implantation experiments. (D) The digital photographs of the subcutaneous implantation area at different time points (the blue arrows indicate the degraded residual gels or sponges) (n = 6). (E) Representative images of H&E staining and Masson staining of tissue in the subcutaneous implantation area at 14 days postoperatively (* indicates the degraded residual material).

The CCK-8 method was used to investigate the effect of extracts with different mass concentrations of GM-Lap@PDA microspheres on the viability of L929 fibroblasts. Following 24-hour co-culture of microsphere extracts with cells, all GM-Lap-treated groups exhibited cell viabilities exceeding 100% across tested concentrations (Figure 4A), potentially attributable to the leaching of Lap. Inorganic ions such as magnesium ions and silica ions released by Lap nanoplatelets in physiological environment would promote the proliferation and migration of cells 61, 62. Notably, the GM-Lap@PDA_II_ group at 10 mg mL^-1^ showed reduced viability compared to GM-Lap and control groups, though remaining above 85%. Importantly, the GM-Lap@PDA_II_ group exhibited time-dependent increases in cell viability. After 48 hours of treatment, cells exposed to various extract concentrations all maintained viabilities > 90%, with the 1.25 mg mL^-1^ group reaching > 138% viability (Figure S12A). The live/dead staining results corroborated the cell viability findings. Fluorescence imaging revealed that L929 fibroblasts from all treatment groups showed green fluorescence and preserved normal spindle morphology after 24-hour exposure to microsphere extracts, comparable to the control group (Figure 4B). Furthermore, all experimental groups demonstrated a various increase in viable cell numbers after 48 hours (Figure S12B). Collectively, these results indicated that GM-Lap@PDA microspheres exhibit favorable cytocompatibility and cell affinity.

The hydrogels and gelatin sponges were subcutaneously injected/implanted into the dorsal region of rats to evaluate material degradation properties and local tissue reactions (Figure 4C). During the two-week observation period, no significant inflammatory reactions (e.g., abscess, ulceration, or tissue fluid exudation) at implantation sites or non-specific toxic reactions (including weight loss, death, or fever) were found (Figure 4D) 18. *In vitro* degradation testing revealed that the GM-Lap@PDA_II_ hydrogel exhibited favorable enzymatic degradation characteristics, achieving over 89% degradation within 7 days (Figure S13A). Consistent with *in vitro* results, the hydrogel showed near-complete degradation by day 14 post-implantation, with no discernible residual material observed. Furthermore, all experimental groups showed progressive weight gain, with the GM-Lap@PDA_II_ group displaying significantly accelerated weight increase (Figure S13B).

Histopathological analysis with H&E and Masson staining revealed that at 7 days postoperatively, the boundary between the gelatin sponge group and the surrounding connective tissue became increasingly indistinct, accompanied by massive cellular infiltration into the interior of the sponge (Figure S14). The GM-Lap@PDA_II_ hydrogel underwent degradation, forming microsphere fragments and exhibiting substantial fusion with the surrounding tissue. At 14 days, the microsphere fragments at the injection site almost completely disappeared, and normal hair bulb and dense collagen fiber bundles could be observed (Figure 4E). To further investigate the inflammatory response of implanted materials in the host and their bioregulatory effects on angiogenesis, a series of immunofluorescence and immunohistochemical staining experiments were conducted (Figures S15A and S16A). During the early inflammatory phase, macrophages play a pivotal role as key immune cells. Substantial evidence indicates that M1 macrophages exacerbate inflammation, while anti-inflammatory M2 macrophages ameliorate the local inflammatory microenvironment and promote tissue regeneration 63. Among the array of pro-inflammatory cytokines, tumor necrosis factor-α (TNF-α), primarily secreted by M1 macrophages at injury sites, is recognized as a major inflammatory mediator 64. Semi-quantitative analysis using ImageJ revealed significantly higher TNF-α expression levels in both gelatin sponge (GS) and hydrogel groups compared to the blank control group (no material implanted) at postoperative day 7, indicating intensified inflammatory responses (Figure S15B). Notably, two weeks after GM-Lap@PDA_II_ hydrogel implantation, the expression levels of M1 phenotype markers (TNF-α and CD86) were downregulated, while that of the M2 phenotype marker (CD206) was upregulated, with no significant difference observed relative to the blank control group (Figure S16B-D). Furthermore, vascular endothelial growth factor (VEGF), a critical angiogenesis-related protein that stimulates endothelial cell proliferation, migration, and vascularization 65, exhibited higher expression levels in the GM-Lap@PDA_II_ group than in both control and gelatin sponge groups at 7 and 14 days postoperatively (Figures S15E and S16E). This enhancement is attributed to the synergistic effects of VEGF secretion by M2 macrophages and sustained release of bioactive ions (Si and Mg) during hydrogel degradation 27. Finally, histopathological analysis of major internal organs after two weeks was performed by H&E staining to assess the systemic toxicity of the GM-Lap@PDA_II_ hydrogel (Figure S17). It was observed that the morphological characteristics of some major organs in the GM-Lap@PDA_II_ group, including heart, liver, spleen, lung, and kidney, were similar to those of the blank group. ​Taken together, these findings demonstrated favorable inherent biodegradability and biocompatibility of the GM-Lap@PDA_II_ microsphere-based hydrogel.

### *In vivo* hemostatic effect

#### Hemostasis on rat tail amputation, liver cruciate incision, liver volumetric defect and liver partial resection models

The above series of characterization and *in vitro* procoagulant assays indicated that GM-Lap@PDA_II_ microspheres/hydrogels have significant potential for hemostasis. Subsequently, the investigations were conducted to assess the hemostatic capacity of the microspheres/hydrogels using four different hemorrhage models in rats: tail amputation, liver cruciate incision, liver volumetric defect and liver partial resection models 11, 42, 66.

For the rat tail amputation model (Figure S18), severe hemorrhage was observed at the incision without any treatment (the blank group), with a total blood loss of 0.353 ± 0.085 g within 772.3 ± 37.6 s. At this time, the bleeding had not stopped and the wound had been oozing blood slightly. When 40 mg of commercial hemostatic agent (YB) was spread on the incision, the bleeding was finally stopped in 192 ± 48.5 s, accompanied by 0.185 ± 0.083 g of blood loss. In contrast, the application of 40 mg of GM-Lap@PDA_II_ cryogel microspheres resulted in the successful cessation of tail incision bleeding in rats within 146.7 ± 20.5 s, accompanied by a notable reduction in blood loss (0.065 ± 0.022 g). The above results demonstrated the great potential of microspheres for rapid hemostasis, which can be used directly as a powder hemostatic agent in open wounds.

**Figure 5:**
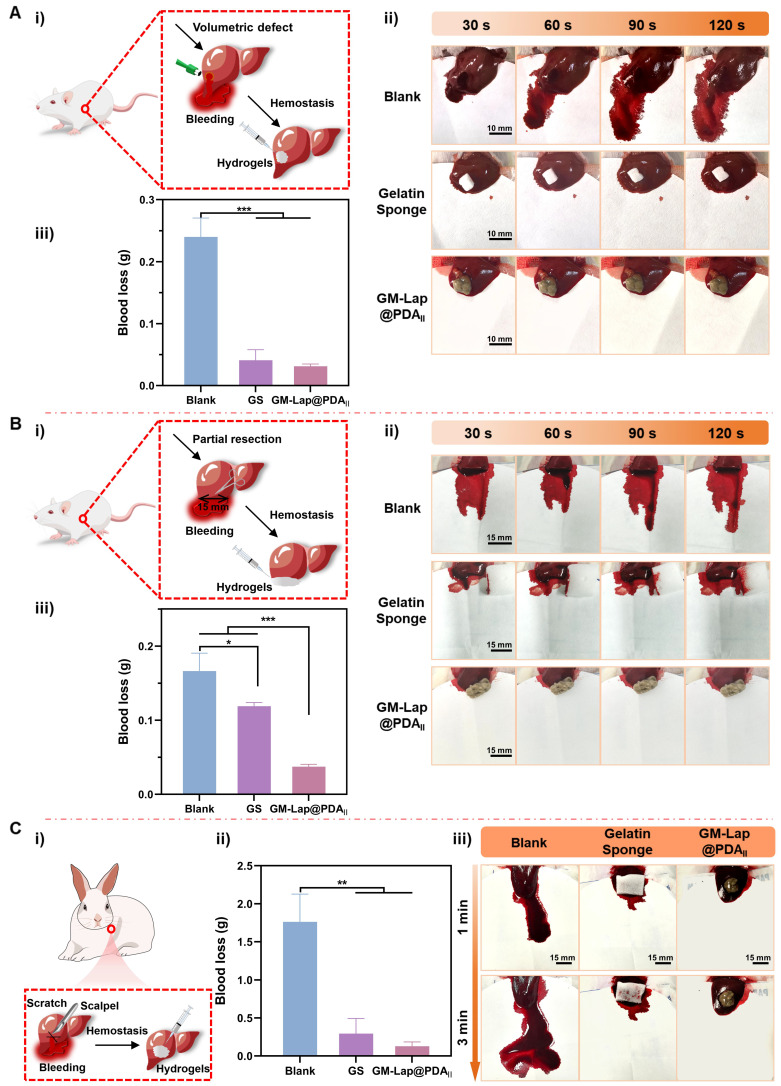
*In vivo* hemostatic effect on rat liver volumetric defect, rat liver partial resection and rabbit liver cruciate incision models. (A) Hemostasis on rat liver volumetric defect: i) Schematic diagram of the rat liver volumetric defect; ii) Representative images of the hemostatic process in each group within 2 min; iii) Cumulative blood loss within 2 min in each group (the blank group, gelatin sponge (GS) and GM-Lap@PDA_II_ hydrogel) (n = 4). (B) Hemostasis on rat liver partial resection: i) Schematic diagram of the rat liver partial resection; ii) Representative images of the hemostatic process in each group within 2 min; iii) Cumulative blood loss within 2 min in each group (the blank group, gelatin sponge and GM-Lap@PDA_II_ hydrogel) (n = 4). (C) Hemostasis on rabbit liver cruciate incision: i) Schematic diagram of the rabbit liver cruciate incision; ii) Cumulative blood loss within 3 min in each group (the blank group, gelatin sponge and GM-Lap@PDA_II_ hydrogel) (n = 3); iii) Representative images of the hemostatic process in each group within 3 min. *p < 0.05, **p < 0.01, and ***p < 0.001.

To evaluate the hemostatic effect of hydrogel for controlling non-compressible intra-abdominal hemorrhage, rat liver cruciate incision, liver volumetric defect and liver partial resection models were established. Due to the brittleness and rich blood supply of the liver 67, the cruciate incision exhibited significant bleeding in the absence of any treatment (the blank group), with a blood loss of 0.205 ± 0.026 g over a two-minute period (Figure S19). In contrast, gelatin sponges, a commercial hemostatic agent, demonstrated a significant reduction in blood loss through absorbing oozing blood (0.013 ± 0.009 g). However, the wounds treated with gelatin sponges did not cease bleeding immediately. Despite comparable blood loss to gelatin sponges, GM-Lap@PDA_II_ hydrogel exhibited a more effective wound sealing effect (0.013 ± 0.008 g) (Movie S4). In the volumetric defect model, the blank group exhibited the highest blood loss (0.240 ± 0.025 g) (Figure 5A). Application of gelatin sponge in the liver defect cavity significantly reduced blood loss to 0.041 ± 0.014 g, attributable to its superior fluid-absorbing capacity. Notably, when GM-Lap@PDA_II_ hydrogel was injected into the defect cavity, the blood loss was further diminished to 0.031 ± 0.003 g within 2 minutes (Movie S5), demonstrating the gel's unique advantage in achieving rapid hemostasis through precise filling of deep and narrow wound cavities. In the partial resection model, it was evident that GM-Lap@PDA_II_ hydrogel could rapidly seal the wound and effectively induce blood coagulation within 30 s (Figure 5B and Movie S6). In comparison to the blank group (0.166 ± 0.020 g) and absorbable gelatin sponge (0.119 ± 0.004 g), the hydrogel group demonstrated a notable reduction in blood loss to 0.037 ± 0.003 g within 2 min. This provided further evidence that the injectable gel has an advantage over the bulk hemostatic agent, in that it allows complete contact to irregularly shaped wounds for rapid hemostasis.

#### Hemostasis on a rabbit liver cruciate incision model

The hemostatic efficacy of GM-Lap@PDA_II_ hydrogel for controlling massive hemorrhage *in vivo* was further evaluated using a rabbit liver cruciate incision model. As illustrated in Figure 5C, the blank group demonstrated severe blood loss (1.762 ± 0.298 g) with persistent oozing observed at the 3-minute time point. In contrast, application of gelatin sponge combined with gentle pressure at the incision site led to a statistically significant reduction in blood loss (0.293 ± 0.163 g). Remarkably, administration of the hemostatic hydrogel into the bleeding site resulted in a further decrease in blood loss (0.128 ± 0.047 g) through physical barrier formation and coagulation-promoting properties, achieving substantial hemostasis at the 3-minute observational endpoint (Movie S7).

#### Hemostasis on a porcine liver rupture model

**Figure 6:**
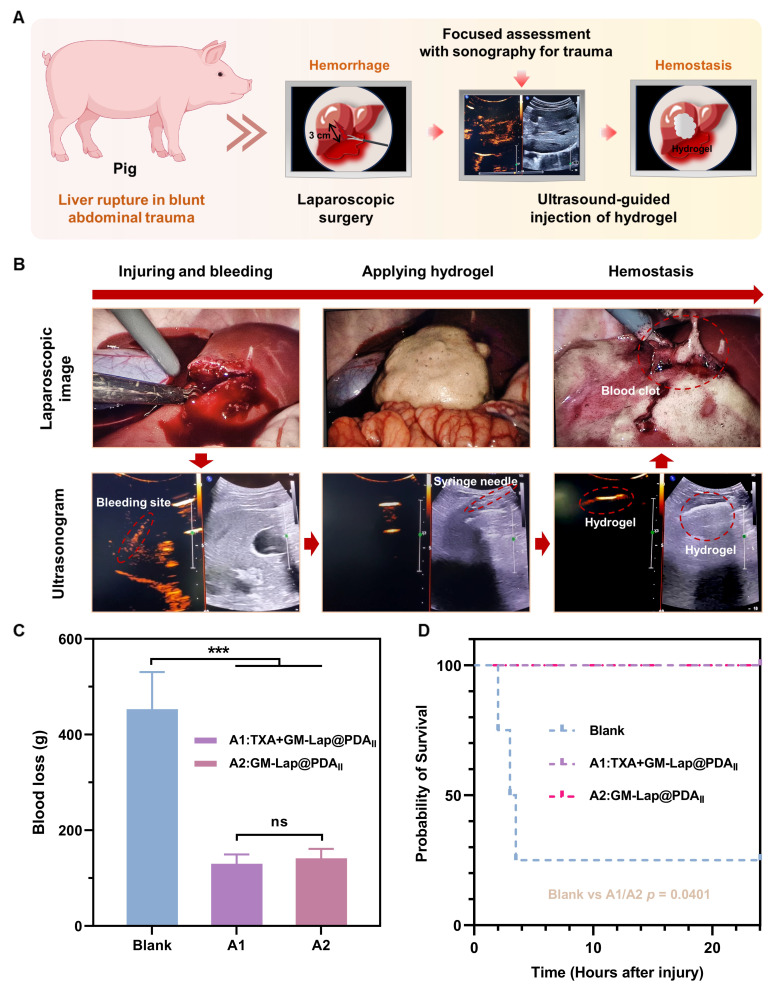
*In vivo* hemostatic effect on a porcine liver rupture model. (A) Schematic diagram demonstrating the application of injectable hemostatic gel in a porcine liver rupture model. Under laparoscopic visualization, a 3-cm-long × 3-cm-deep liver laceration was surgically created at the periphery of the right anterior lobe using laparoscopic scissors. Following pneumoperitoneum evacuation, targeted ultrasound localization identified the hemorrhagic foci. Percutaneous hydrogel administration was performed under real-time ultrasound guidance using a syringe needle, followed by confirmatory laparoscopy at 3 min postprocedural to assess achievement of hemostasis. (B) Sequential intraprocedural laparoscopic and sonographic imaging documenting the continuum from controlled liver laceration generation to hemostatic hydrogel deployment. (C) Cumulative blood loss within T_0_ + 4 h in each group (the blank group, A1 group and A2 group) (n = 4). (D) Kaplan-Meier (K-M) survival curves for each group during the 24-hour observation period (T_0_ + 24 h) (log-rank (Mantel-Cox) test). ***p < 0.001.

Blunt abdominal trauma (BAT) is a relatively common emergency in modern naval warfare, primarily caused by blast shock, hull collision, etc. BAT may induce rupture and hemorrhage of parenchymal organs in the abdominal cavity, and failure to treat it in time may endanger the life of the injured person. For these complex injuries, expeditious and comprehensive diagnosis and treatment are imperative. In this context, the application of highly effective injectable hemostatic materials is critical for successful treatment. Considering that the liver is the most commonly injured organ in blunt abdominal trauma 68, the present study created a liver rupture model under direct laparoscopic vision to simulate hemorrhage from such injuries (Figure 6A). Notably, focused assessment with sonography for trauma (FAST) has been demonstrated in emergency settings to facilitate expeditious assessment of occult hemorrhage by medical professionals 69.

Upon detecting active bleeding, the immediately prepared hemostatic hydrogel was injected under ultrasound guidance into the bleeding site, accomplishing single-organ injury control within 5-8 minutes (Movie S8). Three minutes post-application, laparoscopic ​evaluation of the liver laceration site ​revealed clots formation with observable hemostasis in the experimental group (Figure 6B). Owing to the decline in the modulus at physiological temperature (Figure 2F), and the impact of substantial quantities of blood at the wound, the gel diffused extensively in and around the wound. As time elapsed, the gel interacted with the blood to form a large and stable blood clot adhering to the laceration surface, thereby sustaining hemostasis (Figure S20). At 1 h and 2 h after injury creation (T_0_ + 1 h and T_0_ + 2 h), the abdominal injury site in the experimental group was examined using a portable ultrasound system. The two images revealed comparable effusion depth and spatial distribution, indicating stabilization of the hemorrhagic extent without progressive expansion (Figure S21).

The blood loss during the experimental period served as a key indicator for quantitatively evaluating the hemostatic performance of the injectable microsphere-based hydrogel (GM-Lap@PDA_II_). Tranexamic acid (TXA) is an antifibrinolytic drug that reduces bleeding by inhibiting the enzymatic breakdown of fibrin blood clots. Early pre-hospital use of TXA is effective in reducing mortality associated with trauma-induced hemorrhage 70. As shown in Figure 6C, no statistically significant difference was observed in blood loss between the experimental group A2 (treated with hydrogel only, 141.3 ± 16.9 g) and the experimental group A1 (co-treated with TXA and hydrogel, 130.2 ± 16.6 g). In contrast, the group A2 demonstrated a 69% reduction in blood loss relative to the blank group (453.2 ± 67.3 g). Furthermore, all animals in the hemostatic hydrogel-treated groups (A1 and A2) survived the 24-hour observation period, whereas the blank control group exhibited a precipitous drop to 25% survival (Figure 6D). These findings demonstrated the remarkable efficacy of the hemostatic gel (GM-Lap@PDA_II_) as a first-aid intervention. It is evident that the injectable microsphere-based hydrogel holds considerable potential for application in battlefield trauma care.

## Conclusions

In this work, a novel injectable hydrogel based on mussel-inspired nanocomposite microspheres (GM-Lap@PDA) was developed for non-compressible intra-abdominal hemorrhage control. The cryogel microspheres, fabricated via the W/O emulsification approach followed by gradient freezing, presented a distinct macroporous architecture. The abundant hydrophilic groups synergized with this unique structure to enable the microspheres to rapidly absorb water and blood within a mere 1 second. This superior liquid absorption capacity and negatively charged surface provide great assistance to microspheres in inducing blood coagulation. The hydrogel prepared by hydrating microspheres with sterile normal saline at a specific ratio, exhibited moderate injectability and adjustable mechanical properties that were correlated with the content of DA. Moreover, the presence of the catechol moiety endowed the hydrogel with the capacity to adhere to tissues, thereby facilitating its hemostatic application. *In vitro* coagulation assays and *in vivo* hemostatic experiments in rat liver injury models demonstrated that the hydrogel (GM-Lap@PDA_II_) exhibits excellent procoagulant properties, effectively arresting severe hemorrhage within 2 minutes and resulting in significantly reduced blood loss compared to controls. The ultrasound-guided application of the hydrogel in a porcine liver injury model further confirmed its outstanding emergency hemostatic efficacy. Besides, GM-Lap@PDA_II_ showed favorable biocompatibility and biodegradability. The injectable microsphere-based hydrogel offers dual benefits of convenient portability/storage of microspheres and precise injectable delivery to deep bleeding sites. Collectively, these merits highlight its potential as a frontline hemostatic agent for pre-hospital emergency scenarios.

## Methods

### Materials

Gelatin (~ 250 g Bloom), methacrylic anhydride (MA), lithium phenyl (2,4,6-trimethylbenzoyl) phosphinate (LAP), and dopamine hydrochloride (DA) were purchased from Aladdin (Shanghai, China). Synthetic silicate nanoplatelets (Laponite® XLG-XR, Lap) was obtained from BYK Additives & Instruments. Surfactants (Tween 60 and Span 80) and liquid paraffin were bought from Sinopharm (China). Fetal bovine serum (FBS), DMEM (high glucose) cell culture medium, penicillin-streptomycin solution (PS) and phosphate buffered saline (PBS) were provided by Bio-Channel Biotechnology Co., Ltd (China). The cell counting kit-8 (CCK-8 kit) and Fluorescein diacetate/Propidium Iodide (FDA/PI) stain were purchased from Jiangsu KGI Biotechnology Co., Ltd (China) and Sigma-Aldrich (USA), respectively. All biochemical reagents were used as received. Fresh porcine skins were bought at a local market. Commercial Yunnan Baiyao powder and absorbable gelatin sponge were obtained from Yunnan Baiyao Group Co., Ltd (China) and Xiangen Medical Technology Development Co., Ltd (China), respectively.

### Synthesis of GelMA

GelMA was synthesized by reacting gelatin with methacrylic anhydride as previously described 71. Gelatin (20 g) was completely dissolved in PBS buffer solution (0.01 M, pH = 7.2 ~ 7.4, 200 mL) at 50 °C. Methacrylic anhydride (12.8 g) was then added to the above gelatin solution dropwise (200 µL min^-1^) with a syringe pump, and the reaction was stirred at 50 °C for more than 3 h. The reacted solution was dialyzed in ultrapure water for 7 days (MWCO: 14000 Da) to remove some unreacted low molecular weight compounds and potentially cytotoxic by-products. The dialysate was freeze-dried to obtain a white sponge-like product, which was stored at -20 °C for later use. The methacrylation of gelatin was proved using ^1^H-nuclear magnetic resonance (^1^H-NMR) spectroscopy (AVANCE III HD 600MHz, Bruker, USA). The quantification of the degree of methacrylation (DM) of gelatin was calculated according to the reported method 72.

### Preparation of GM-Lap@PDA cryogel microspheres

Laponite® XLG-XR (225 mg) and DA were simultaneously dispersed in 7.5 mL of ultrapure water and stirred vigorously at room temperature for more than 5 h to obtain a dark viscous liquid (Lap@PDA). GelMA (1.5 g), LAP (37.5 mg), and Tween 60 (150 mg) were dissolved in deionized water at 50 °C to form a 20 wt% solution. The above aqueous solution was mixed with the Lap@PDA solution in equal volumes, then stirred at high speed for 10 min and sonicated to remove foam to obtain a homogeneous solution. This aqueous solution was added dropwise to liquid paraffin (150 mL) containing Span 80 (300 mg) with continuous stirring at 450 rpm for 20 min to form a W/O emulsion. The emulsion was incubated at 4 °C for 20 min, followed by -20 °C for 30 min, before transfer to -196 °C for final freezing.​ The frozen emulsion was irradiated under UV light for 5 min before the oil phase was washed with acetone and the microspheres were collected. The microspheres were named GM-Lap, GM-Lap@PDA_I_ and GM-Lap@PDA_II_ based on the mass of DA (0 mg, 6 mg and 12 mg), respectively.

### Characterizations of Lap@PDA and GM-Lap@PDA cryogel microspheres

#### FE-SEM

The morphology of Lap@PDA and GM-Lap@PDA cryogel microspheres was observed using a field-emission scanning electron microscope (FE-SEM, Ultra Plus, Zeiss, Germany) at an accelerating voltage of 1-2 kV. At the same time, the elemental compositions of Lap and Lap@PDA were analyzed by element mapping and energy dispersive spectroscopy (EDS).

#### FT-IR

The chemical structure of Lap@PDA and GM-Lap@PDA cryogel microspheres was determined using fourier transform infrared spectroscopy (FT-IR, VERTEX 70, Bruker, USA) with a scan wavenumber range of 4000 to 400 cm^-1^.

#### XRD

XRD patterns of Lap and Lap@PDA were obtained by using an X-ray diffractometer (XRD, Smartlab, Rigaku, Japan) by operating at a voltage of 3 kV and employing Cu Kα filtered radiation (λ = 1.5406 nm).

#### Zeta potential

Zeta potential of the microspheres was measured with a zeta potential analyzer (Zetasizer Nano ZS90, Malvern, UK). All the samples were diluted to the concentration of 5 mg mL^-1^ (n = 3).

#### Absorption capacity study of microspheres

The absorption capacity of GM-Lap@PDA microspheres was determined using an optical surface analyzer (OSA200, China) with the sessile drop method. 5-10 mg of microspheres were flattened on a glass plate, and the absorption of the microspheres into aqueous solution or anticoagulated whole blood (3.8% sodium citrate/blood, 1/9 v/v) within 1 s was captured using dynamic contact angle measurement software (n = 3). The dried microspheres (W_0_) were immersed in saline to make them completely swell and then the wet microspheres were weighed (W_i_) after removing their surface water with filter paper (n = 3). The swelling ratio was calculated by the following formula: Swelling ratio (%) = (W_i_-W_0_) / W_0_ × 100.

### Preparation of injectable microsphere-based hydrogels

The microspheres (100 mg) and sterile saline (300 µL) were loaded into two syringes connected by Luer taper and the syringes were rapidly pushed back and forth until a homogeneous injectable gel was obtained. The pre-gel can be injected through a 20 G needle.

### Characterization of injectable microsphere-based hydrogels

#### Injection force measurement

The injection force of the hydrogels was measured using a universal testing machine (WDW-5, KECETEST, China) in compression mode. A syringe (2.5 mL) equipped with a 20 G needle (Φ = 0.9 mm, L = 37 mm) containing the pre-gel was fixed to the universal testing machine, and the compressive plate pressed down the syringe plunger at a rate of 2 mL min^-1^. The test was stopped when the pressure stabilized, and the pressure was recorded as the injection force (n = 3).

#### TGA

Thermal stability of hydrogels was determined using a thermogravimetric analyzer (TGA, TG209 F3, NETZSCH, Germany) under nitrogen (N_2_) atmosphere with a warming rate of 20 °C min^-1^ in the temperature range from 30 to 600 °C.

#### Rheological measurement

Rheological tests of hydrogels were performed at room temperature on a rheometer (MCR302, Anton Paar, Austria) equipped with parallel plates. Strain amplitude sweeps (0.1%-1000%) were performed at a constant angular frequency (10 rad s^-1^). Temperature scans (10-60 °C) were conducted at a fixed strain and frequency (0.1%, 10 Hz) with a warming rate of 5 °C min^-1^. The shear thinning behavior (shear rate: 1-100 s^-1^) of hydrogels was characterized by measuring the linear viscosity (η) at a constant strain (1%).

#### Lap shear test

The lap shear test of hydrogels was conducted following the American Society of Testing Materials (ASTM) F2255 standard. The glass slides (75 mm × 25 mm) and fresh porcine skin (40 mm × 20 mm) treated with ultrapure water and PBS were used as adhesion substrates. The pre-gel (100 μL) was injected between two glass slides with a lap area of 625 mm^2^ (25 mm × 25 mm) for the adhesive joint (n = 4). To minimize the effect of tissue deformation during stretching on the test results, one side of the fresh porcine skin was adhered to the rigid backing using cyanoacrylate glue. Subsequently, 100 μL of pre-gel was injected between two clean or blood-coated porcine skin with a lap area of 400 mm^2^ (20 mm × 20 mm) (n = 4). All tests were performed using a universal testing machine at a tensile speed of 5 mm min^-1^. The lap shear adhesion strength was defined as the ratio of the maximum tensile force at adhesive joint failure to the lap area.

### *In vitro* degradation behavior study

The freshly prepared hydrogel (W_0_) was completely submerged in PBS with collagenase type II (10 mg mL^-1^) (n = 4). After incubation at 37 °C for some time, the residual sample was weighed (W_i_) after removing excess water from their surface with dry filter paper. The degradation ratio of the hydrogel was calculated according to the following equation: Degradation ratio (%) = (W_0_-W_i_) / W_0_ × 100.

### *In vitro* blood-clotting performance test

#### Whole blood clotting time

After incubating 10 mg of the sample (blank, GM-Lap@PDA cryogel microspheres and Yunnan Baiyao powder) for 5 min at 37 °C, 500 μL of anticoagulated whole blood (3.8% sodium citrate/blood, 1/9 v/v) was mixed with it and further incubated at 37 °C for 3 min. Subsequently, 250 µL of CaCl_2_ (0.025 M) was rapidly added to the above mixture to trigger coagulation and start the timer. Clot formation was observed every 10 s (n = 4).

#### Whole blood clotting kinetics

10 mg of the sample (blank, GM-Lap@PDA cryogel microspheres and Yunnan Baiyao powder) was placed in a 24-well plate and incubated at 37 °C for 5 min. Subsequently, 200 µL of anticoagulated whole blood and 20 µL of CaCl_2_ solution (0.1 M) were added to each well, and the mixture was incubated at 37 °C for 3 min. Then the uncoagulated blood cells were rinsed off using 3 mL of ultrapure water, following incubation on a shaker (40 rpm) for 10 min. Eventually, the absorbance of the supernatant at 540 nm was measured using a microplate reader (HBS-1096A, DeTie, China) (n = 3). Blood coagulation index (BCI) was calculated by the formula: BCI (%) = A_sample_ / A_blank_ × 100

The sample (blank, 200 µL of the hydrogel and 67 mg of gelatin sponge), 200 μL of anticoagulated whole blood, and 20 µL of CaCl_2_ solution (0.1 M) were co-incubated at 37 °C in a 24-well plate. After incubation for 0.5 min, 1 min, 2 min and 3 min, the uncoagulated blood cells were hemolyzed with 3 mL of ultrapure water, and then following incubation on a shaker (40 rpm) for 10 min. Eventually, the absorbance at 540 nm of the resulting hemoglobin solution was measured (n = 3).

#### Thromboelastograph analysis

Following the injection of 100 μL of gel (GM-Lap@PDA_II_) into each EP tube (1.5 mL), the addition of 1 mL of anticoagulated whole blood and subsequent vortexing for 30 s were required. The resultant mixture was then incubated at 37 °C for 10 min, after which the vortexing was continued for a further 30 s. The mixed whole blood solution (340 µL) and CaCl₂ solution (20 µL, 0.2 M) were then added to the test cup and the whole dynamic coagulation process was monitored by thromboelastograph meter (CFMS LEPU-8800, China). The whole blood solution that did not come into contact with the sample and gelatin sponge groups were used as blank and positive controls, respectively (n = 3).

#### Blood cell adhesion

The sample (15 mg of microspheres/200 μL of hydrogel) was completely submerged in anticoagulated whole blood (500 μL/1 mL) and incubated at 37 °C for 30 min. Subsequently, unadhered blood cells were gently rinsed three times with PBS, and then all samples were immersed in 2.5% glutaraldehyde fixative for 2 h. After that, blood cells were stepwise dehydrated using 25%, 50%, 75%, and 100% ethanol solutions with time interval of 15 min. Finally, the samples were naturally air-dried and observed using FE-SEM. Platelet-rich plasma (PRP) was obtained by centrifuging anticoagulated whole blood at 1200 rpm for 15 minutes. Other steps were similar to those referred to as above.

#### APTT and PT

Platelet-poor plasma (PPP) was obtained by centrifuging anticoagulated whole blood at 3000 rpm for 15 minutes. Microspheres (20 mg) were thoroughly mixed with PPP (1 mL), and 50 μL of the mixture was incubated with pre-warmed APTT reagent (50 μL) at 37 °C for 5 minutes. After incubation, 50 μL of pre-warmed CaCl₂ solution (0.025 M) was added to initiate coagulation, and the plasma clotting time (APTT) was measured using a semi-auto coagulation analyzer (C2000-4, Mindray, China). For PT determination, 50 μL of the microsphere-PPP mixture was incubated at 37 °C for 3 minutes, followed by addition of 100 μL pre-warmed PT reagent, with subsequent recording of the plasma coagulation time (PT) (n = 3).

### *In vitro* biocompatibility

#### Hemolysis assay

Erythrocytes were obtained by centrifuging anticoagulated whole blood at 250 *g* for 15 minutes. Subsequently, the erythrocytes were washed several times with PBS until the supernatant was clarified, and then diluted with PBS to form a 5% (v/v) suspension. The sample (200 μL of hydrogel) was placed in a 24-well plate and the erythrocyte suspension (1 mL) was added. The 5% (v/v) solution obtained by mixing 0.1% of Triton X-100 with erythrocytes and the erythrocyte suspension without sample treatment were used as positive and negative controls, respectively. The groups were incubated at 37 ℃ for 1 h at a shaking speed of 80 rpm, and then the erythrocyte suspension was centrifuged at 2000 *g* for 15 min. Eventually, the absorbance at 540 nm of the supernatant was measured (n = 3). The hemolysis ratio was calculated by the formula: Hemolysis ratio (%) = (A_experiment_-A_negative_) / (A_positive_-A_negative_) × 100

#### Cytotoxicity test

The cytotoxicity of GM-Lap@PDA microspheres to L929 cells was evaluated by CCK-8 assay. ​​After soaking microspheres sterilized with 75% ethanol in growth medium at 37°C for 24h, the medium was filtered through 0.45µm and 0.22µm membranes sequentially to obtain the extraction solution. L929 cells were seeded in a 96-well plate at a density of 3000 cells per well. Following incubation at 37 °C in 5 % CO_2_ atmosphere for 24 h, the growth medium was removed from the 96-well plate and a series of microspheres extractions with a concentration gradient (10, 5, 2.5, 1.25 mg mL^-1^) were added (duplicated samples, n = 5). After incubating L929 cells at 37 °C for 24 h or 48 h, the extract in each well was aspirated and 100 µL of a mixture containing 90 µL of DMEM and 10 µL of CCK-8 reagent was supplemented. 2 h later, the absorbance of each well at 450 nm was measured using a microplate reader. The wells in which the cells were not co-incubated with the sample extracts and the wells in which there were no cells but only DMEM and CCK-8 reagent were set up as blank control and solvent groups, respectively. The cell viability was calculated according to the following formula: Cell viability (%) = (A_experiment_-A_solvent_)/(A_control_-A_solvent_) × 100

Dual-fluorescence viability using FDA/PI stain was conducted to further assess the cytocompatibility of GM-Lap@PDA microspheres to L929 cells. After incubating L929 cells at 37 °C for 24 h or 48 h, the extract in each well was removed and 100 µL of a mixture containing 94.5 µL of PBS, 5 µL of PI and 0.5 µL FDA was added (duplicated samples, n = 4). Following incubation for 5 min away from light, the fluorescent stain was discarded and PBS was supplemented to each well. The live/dead L929 cells were observed with an inverted fluorescence microscope (MF52, Mshot, China).

### *In vivo* biocompatibility

The biocompatibility *in vivo* of the hydrogel was assessed by subcutaneous implantation test on the dorsal region of SD rats (200 ~ 220 g, male, n = 6). The animal care and study followed the Guidelines for Care and Use of Laboratory Animals of Southeast University in China and approved by the Animal Ethics Committee of Southeast University in China (No. 20240529010). After shaving the dorsal fur of anesthetized rat, the epidermis was cut on both sides of the dorsal spine with surgical scissors (the length of the incision was 10~15 mm) to prepare a subcutaneous pocket, in which sterilized absorbable gelatin sponge (10 mm in length and width, and 2 mm in thickness) and GM-Lap@PDA_II_ hydrogel (freshly prepared from UV-sterilized microspheres and sterile saline, 100 μL) were implanted/injected, respectively. Subcutaneous pocket without any implant was set as blank control group. A maximum of three implants were placed in each animal and it was ensured that each subcutaneous pocket was at least 2 cm apart. After surgical wound closed, the animals were allowed to recover from anesthesia. At 3, 7 and 14 days after implantation, the weight of the animals was recorded and the animals were euthanized by over anesthesia. Peri-implant subcutaneous tissues and visceral organs (heart, liver, spleen, lung, and kidney) were taken, fixed overnight in 4% paraformaldehyde solution, and processed through paraffin embedding. The tissue samples underwent hematoxylin and eosin (H&E), Masson's trichrome, immunohistochemical (TNF-α), and immunofluorescence (CD86, CD206 and VEGF) staining for comprehensive histopathological evaluation.

### *In vivo* hemostatic performance assay

The animal care and study followed the Guidelines for Care and Use of Laboratory Animals of Southeast University in China and approved by the Animal Ethics Committee of Southeast University in China (No. 20240529010, No. SEU-IACUC-20250404001 and No. SEU-IACUC-20250416002).

#### Hemostasis on rat tail amputation

SD rats (200 ~ 220 g, male, n = 4) were randomly divided into three groups and fed normally for 5-7 days. After the rats were anesthetized with isoflurane, the distal 6 cm of the tail was excised. The incision was allowed to bleed naturally for 5 s. The effluent blood was wiped off with sterile gauze and a weighed filter paper was placed under the incision. 40 mg of hemostatic material (GM-Lap@PDA_II_ microspheres and Yunnan Baiyao powder) was immediately placed over the trauma with normal bleeding and timing was started. Hemostasis time was recorded when the incision surface was free of bleeding, at which point the filter paper was removed and weighed. The incision without sample treatment was set up as a blank control group.

#### Hemostasis on rat liver cruciate incision

SD rats (200 ~ 220 g, male, n = 4) were anesthetized, after which the abdominal cavity was opened to expose the liver, ​and a cruciate incision (10 mm in length and 2-3 mm in depth) was created on the right lobe of the liver using a scalpel.​ After the wound bled naturally for 3 s, the effluent blood was wiped off with sterile gauze, and a weighed pad of filter paper was placed under the liver. Immediately, 200 μL of GM-Lap@PDA_II_ hydrogel was injected into the bleeding site, and photographs were taken at 30 s intervals to record the bleeding of the trauma. The filter paper was removed and weighed at 2 min to calculate the blood loss. The blank group without sample treatment and the absorbable gelatin sponge group (type B, 20 mm × 20 mm) were used as controls.

#### Hemostasis on rat liver volumetric defect

SD rats (200 ~ 220 g, male, n = 4) were anesthetized, the abdominal cavity was opened to expose the liver, and a volumetric defect (diameter: 6 mm, depth: 5 mm) was created in the left lobe of the liver using a biopsy punch followed by a surgical scissor. After 3 seconds of free bleeding, the blood was gently blotted with sterile gauze, and 200 μL of GM-Lap@PDA_II_ hydrogel was immediately applied to the bleeding site. Digital images were captured at 30-second intervals to monitor hemostasis progression. At 2 minutes post-application, the blood loss was quantified by weighing filter papers. The blank group without sample treatment and the absorbable gelatin sponge group (dimension: 10 mm × 5 mm × 5 mm) were used as controls.

#### Hemostasis on rat liver partial resection

SD rats (200 ~ 220 g, male, n = 4) were anesthetized, the abdominal cavity was opened to expose the liver, and a 15 mm segment of tissue was excised from the left lobe using a surgical scissor. After the wound bled naturally for 3 s, the effluent blood was wiped off with sterile gauze, and a weighed filter paper was placed under the liver. Immediately, 300 μL of GM-Lap@PDA_II_ hydrogel was injected into the bleeding site, and photographs were taken at 30 s intervals to record the bleeding of the trauma. The filter paper was removed and weighed at 2 min to calculate the blood loss. The blank group without sample treatment and the absorbable gelatin sponge group (type B, 20 mm × 20 mm) were used as controls.

#### Hemostasis on rabbit liver cruciate incision

New Zealand White rabbits (2.5-3 kg, male, n = 3) were anesthetized with isoflurane, the liver was exposed through an abdominal incision, and excess plasma around the liver was carefully removed with sterile gauze. A pre-weighed piece of filter paper was placed underneath the liver and subsequently, a cruciate incision (15 mm in length and 2-3 mm in depth) was made in the ​in the left lobe of the liver with a scalpel. Immediately after gently wiping away blood from the wound, 500 μL of GM-Lap@PDA_II_ hydrogel was injected *in situ* into the bleeding site. The filter paper was removed and weighed at 3 min to calculate the blood loss in each group. The blank group without sample treatment and the absorbable gelatin sponge group (type A, 30 mm × 20 mm) were used as controls.

#### Hemostasis on porcine liver rupture

Twelve experimental white pigs (25-30 kg, male, n = 4) were randomly divided into three groups: a blank control group (conventional resuscitation), experimental group A1 (novel protocol: tranexamic acid (TXA) + GM-Lap@PDA_II_ hydrogel), and experimental group A2 (identical protocol without TXA). Following anesthesia, a laceration of 3 cm in length and 3 cm in depth was surgically created on the lateral margin of the right anterior liver lobe using laparoscopic scissors under direct visualization (KARL STORZ, Germany). The time of injury establishment was designated as T_0_.

Ultrasound guidance (Ultrasonic Color Doppler Diagnostic system, Philips EPIQ Elite, China) was used to percutaneously localize the bleeding site, followed by injection of hemostatic hydrogel for uniform wound coverage. Hemostasis was assessed laparoscopically ~3 minutes post-injection. Vital signs were continuously monitored, with portable ultrasound (Vscan Air CL, GE HealthCare, USA) screening for active bleeding. At T₀ + 4 hours, total blood loss was quantified by aspirating intra-abdominal blood/clots and absorbing residual free blood with gauze. Wounds were sutured, anesthesia terminated, and animals resuscitated. (Detailed methodology is provided in Supplementary Material).

### Statistical analysis

All the experiments were repeated for at least three times and the data were expressed as mean ± standard deviation (SD). Unpaired Student's t test (two-tailed) or One-way analysis of variance (ANOVA) test followed by Tukey's test was performed for statistical analysis (GraphPad Prism 9.5.0). *p* < 0.05 was considered statistically significant.

## Supplementary Material

Supplementary figures and movie legends.

Supplementary movie 1.

Supplementary movie 2.

Supplementary movie 3.

Supplementary movie 4.

Supplementary movie 5.

Supplementary movie 6.

Supplementary movie 7.

Supplementary movie 8.

## Abbreviations

GelMA: gelatin methacryloyl
PDA: polydopamine
NCIAH: non-compressible intra-abdominal hemorrhage
Lap: Laponite
DA: dopamine
W/O: water-in-oil
APTT: activated partial thromboplastin time
PT: prothrombin time
WBCT: whole blood clotting time
TGA: thermogravimetric analysis
BCI: blood coagulation index
TEG: thromboelastogram
R: reaction time
K: clot formation time
A: angle
MA: maximum amplitude
CI: coagulation composite index
TNF-α: tumor necrosis factor-α
VEGF: vascular endothelial growth factor
BAT: blunt abdominal trauma
FAST: focused assessment with sonography for trauma
TXA: tranexamic acid
^1^H-NMR: ^1^H-nuclear magnetic resonance
DM: degree of methacrylation
FE-SEM: field-emission scanning electron microscope
EDS: energy dispersive spectroscopy
FT-IR: fourier transform infrared spectroscopy
XRD: X-ray diffractometer
PPP: platelet-poor plasma
PRP: platelet-rich plasma
H&E: hematoxylin and eosin
