# Supplementary material for: Injectable hydrogels based on mussel-inspired nanocomposite microspheres for non-compressible intra-abdominal hemorrhage control
Source: Theranostics. 2025 Jul 28;15(16):8509–30. doi: 10.7150/thno.118901 (PMC12374639; doi:10.7150/thno.118901)
Supplement: Supplementary file 1 — Supplementary figures and movie legends. [file thnov15p8509s1.pdf]

Supplementary Material

Injectable hydrogels based on mussel-inspired nanocomposite microspheres for non-compressible intra-abdominal hemorrhage control

Tao Liu[1,*], Peng Ma[2,*], Fengya Jing[1], Yinghua Tao[1], Jinfang Lu[2], Dandan Wei[1], Liuxin Yang[1], Feiling Feng[3], Yonghua Li[2], Hongbin Yuan[2], Tianzhu Zhang[1,4]

1. State Key Laboratory of Digital Medical Engineering, School of Biological Science and Medical Engineering, Southeast University, Nanjing 210096, China
2. Department of Anesthesiology, Changzheng Hospital, Second Affiliated Hospital, Naval Medical University, Shanghai 200003, China
3. Department of Biliary Tract Surgery I, Third Affiliated Hospital, Naval Medical University, Shanghai 200438, China
4. Advanced Ocean Institute of Southeast University, Nantong 226010, China

[*] These authors contributed equally to this work.

Corresponding authors: Tianzhu Zhang, email: zhangtianzhu@seu.edu.cn; Hongbin Yuan, email: jfjczyy@aliyun.com; Yonghua Li, email: liyonghua1207@126.com; Feiling Feng, email: ffeiling@163.com.

This file includes: Figures S1-S21 and Movies S1-S8

Hemostasis on porcine liver rupture

Twelve experimental white pigs (25-30 kg, male, n = 4) were randomly divided into three groups: the blank control group with a conventional fluid resuscitation and antishock protocol; experimental group A1 with a novel fluid resuscitation and antishock protocol consisting of tranexamic acid (TXA) and hemostatic gel (GM-Lap@PDA$_{II}$); and experimental group A2, with the same rescue protocol as in experimental group 1, except that no TXA was used.

The experimental pigs were sedated with intramuscular injections of atropine and Su-Mian-Xin, and then anaesthetized intravenously with peripheral intravenous sequential infusions of propofol, tilidine and vecuronium bromide. The depth of anaesthesia was subsequently maintained by inhalation of isoflurane following muscle relaxation. Invasive arterial blood pressure monitoring was established via the femoral artery, blood oxygen saturation and pulse were measured via tongue, and arterial blood was drawn intermittently for blood gas analysis.

After successful anaesthesia, standard laparoscopic perforation for liver surgery was performed in the abdomen of the experimental pig. Following the establishment of an artificial pneumoperitoneum, a laceration of 3 cm in length and 3 cm in depth was created with laparoscopic scissors on the lateral margin of the right anterior lobe of the liver under direct laparoscopic visualization (KARL STORZ, Germany). Subsequently, the laparoscope was withdrawn and the moment of injury creation was recorded as T_0 time.

After locating the bleeding site in the liver under ultrasound guidance (Ultrasonic Color Doppler Diagnostic system, Philips EPIQ Elite, China), the injection needle was used to puncture the skin to the most significant point of bleeding, and the hemostatic hydrogel (20 mL) was injected uniformly so that the gel covered as completely as possible the liver wounds of the experimental animals. Following a period of approximately 3 min, the bleeding of the wound was observed through laparoscopy. After taking pictures for documentation, the laparoscope was removed, and the blood pressure and heart rate of the experimental animal were continuously monitored. The portable ultrasound system (Vscan Air CL, GE HealthCare, USA) was held and used to

detect whether there was further active bleeding in the trauma area.

At T_0 + 4 h, the laparoscope was employed to access the abdominal region, where a meticulous examination of the wounds was conducted in each group. Subsequently, an aspirator was utilized to aspirate blood and clots, and gauze was employed to absorb the residual free blood and calculate the total blood loss during the procedure. Finally, the laparoscope was withdrawn from the body, and the wound was sutured before the anesthesia was discontinued and the experimental animals were resuscitated.

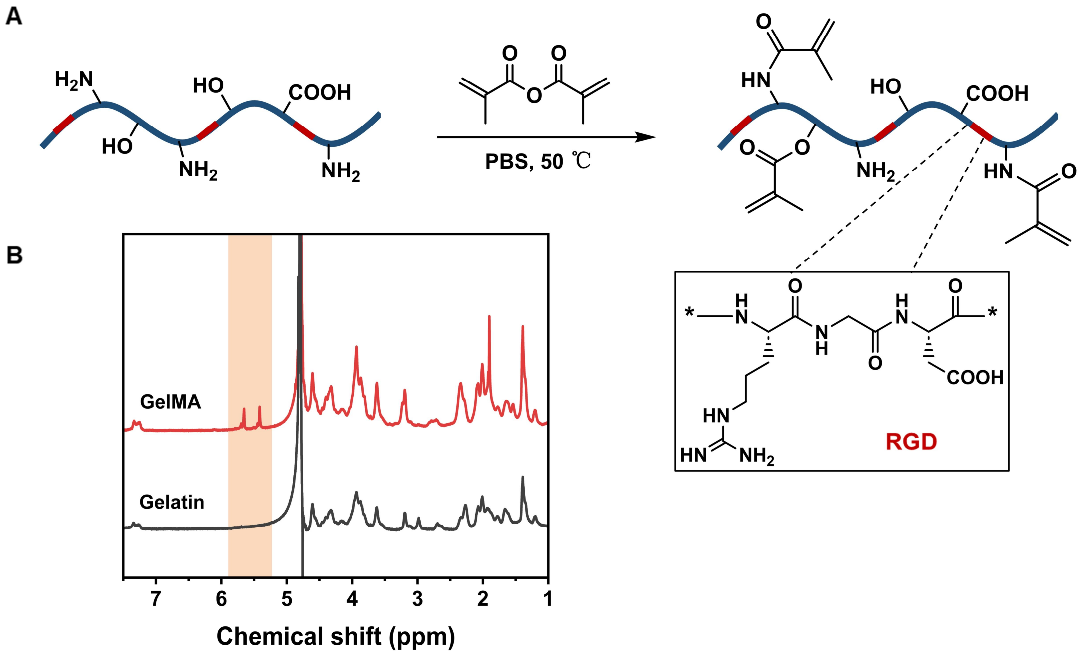

Figure S1. (A) The synthesis mechanism of GelMA, and RGD represents the arginine-glycine-aspartic acid sequence. (B) ^{1}H-NMR spectra of gelatin and GelMA.

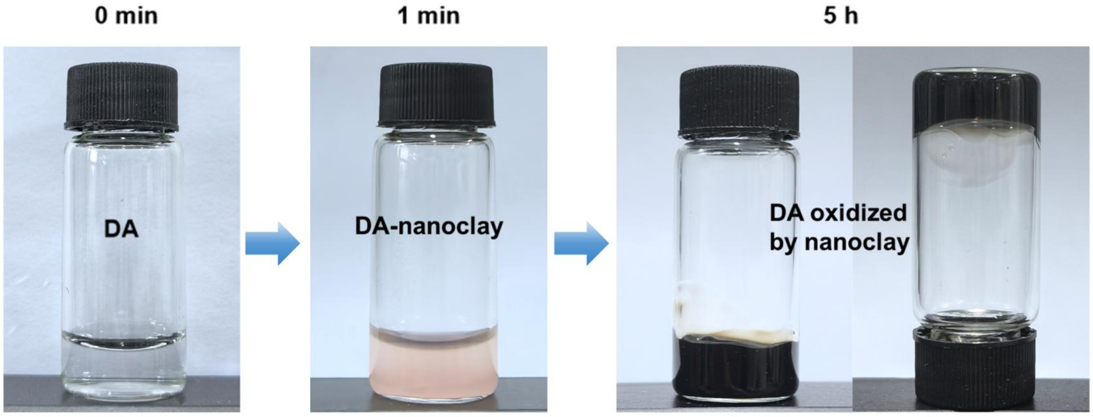

Figure S2. Representative images of the process of *in situ* oxidation of DA in nanoclay (Lap) solutions.

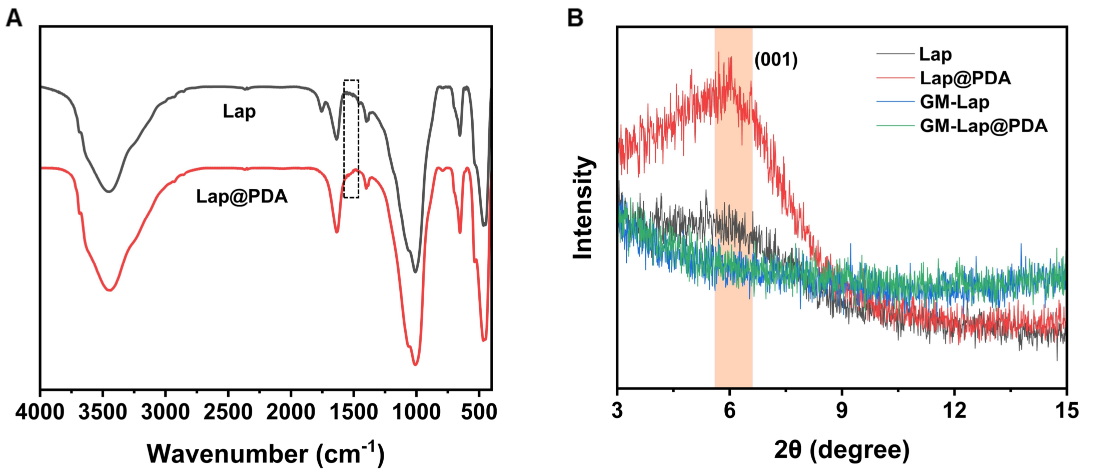

Figure S3. (A) FT-IR spectra of Lap and Lap@PDA. (B) XRD patterns of Lap and Lap@PDA.

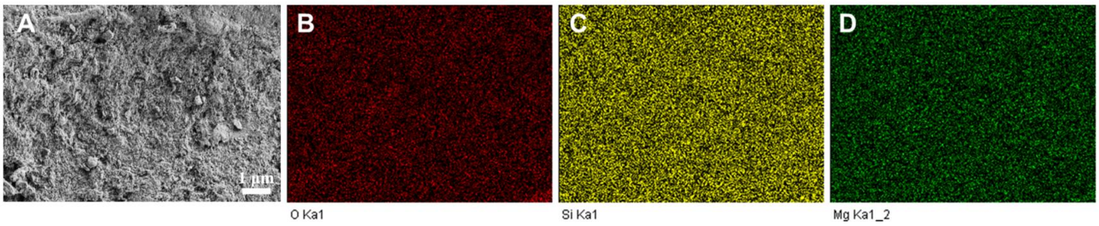

Figure S4. (A) SEM image of Lap nanoplatelets. (B-D) SEM-EDS elemental mapping of O, Si and Mg.

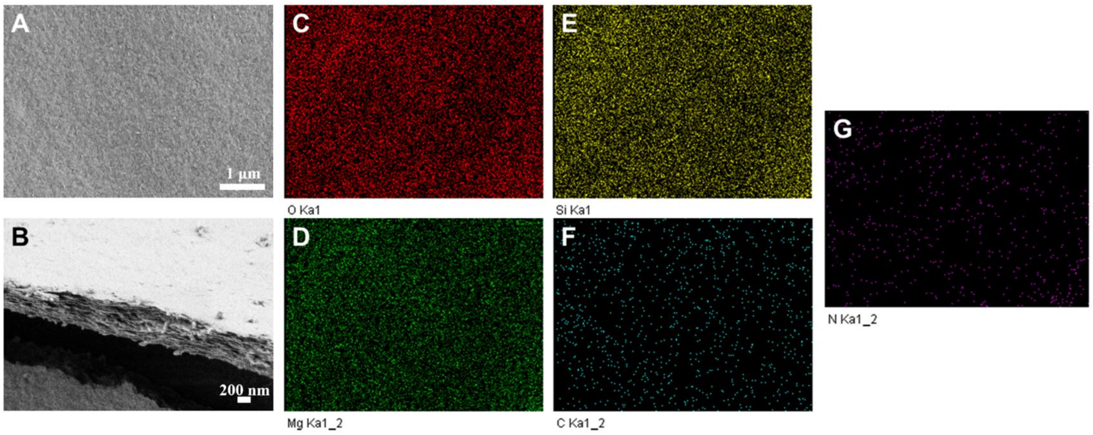

Figure S5. (A, B) SEM images of Lap@PDA. (C-G) SEM-EDS elemental mapping of O, Mg, Si, C and N.

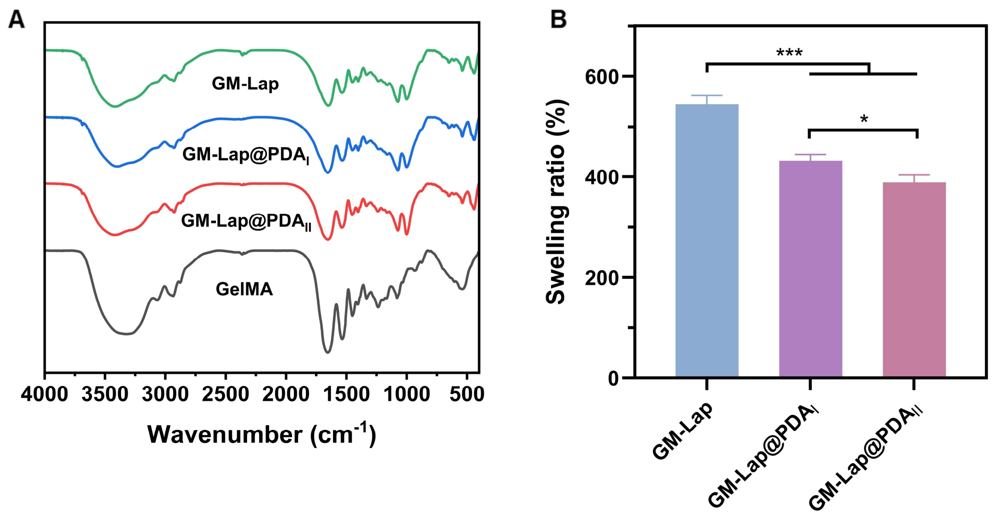

Figure S6. (A) FT-IR spectra of GelMA and GM-Lap@PDA microspheres. (B) Swelling ratios of GM-Lap@PDA microspheres (n = 3). **p* < 0.05 and ****p* < 0.001.

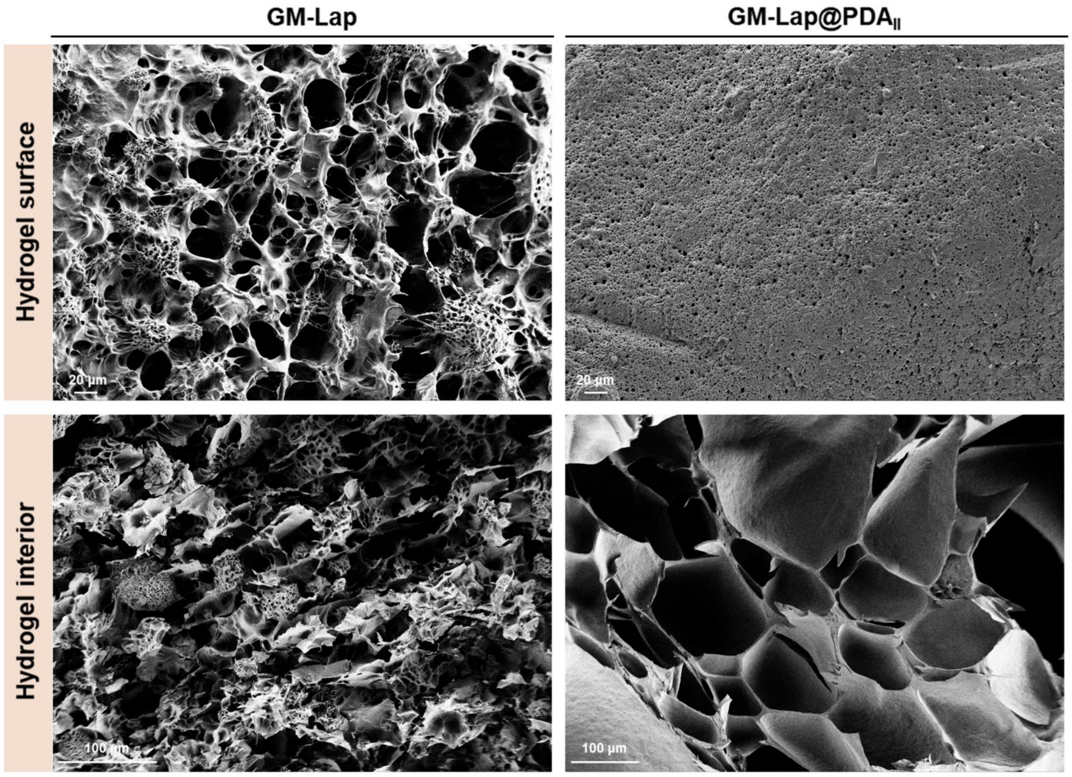

Figure S7. The morphology images of GM-Lap and GM-Lap@PDA$_{II}$ hydrogels. The GM-Lap system exhibited loosely connected microsphere fragments with clearly defined, isolated boundaries, a structure resulting from non-covalent interactions between the photocrosslinked GelMA skeleton and Laponite, while lacking robust inter-microsphere connections. In contrast, the GM-Lap@PDA system displayed a highly interconnected porous structure, where the original microspheres completely lost their distinct morphology after hydration. This difference confirmed that PDA triggers a profound fusion of the microspheres.

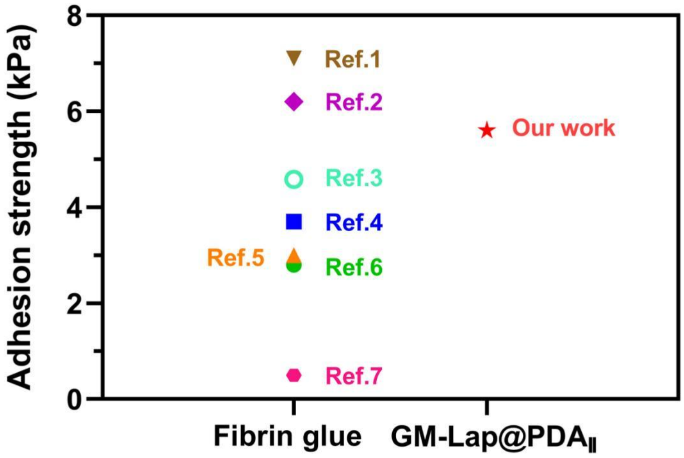

Figure S8. Comparison of lap-shear adhesion strength for commercial fibrin glue reported in the literatures and GM-Lap@PDAII hydrogel. [1-7]

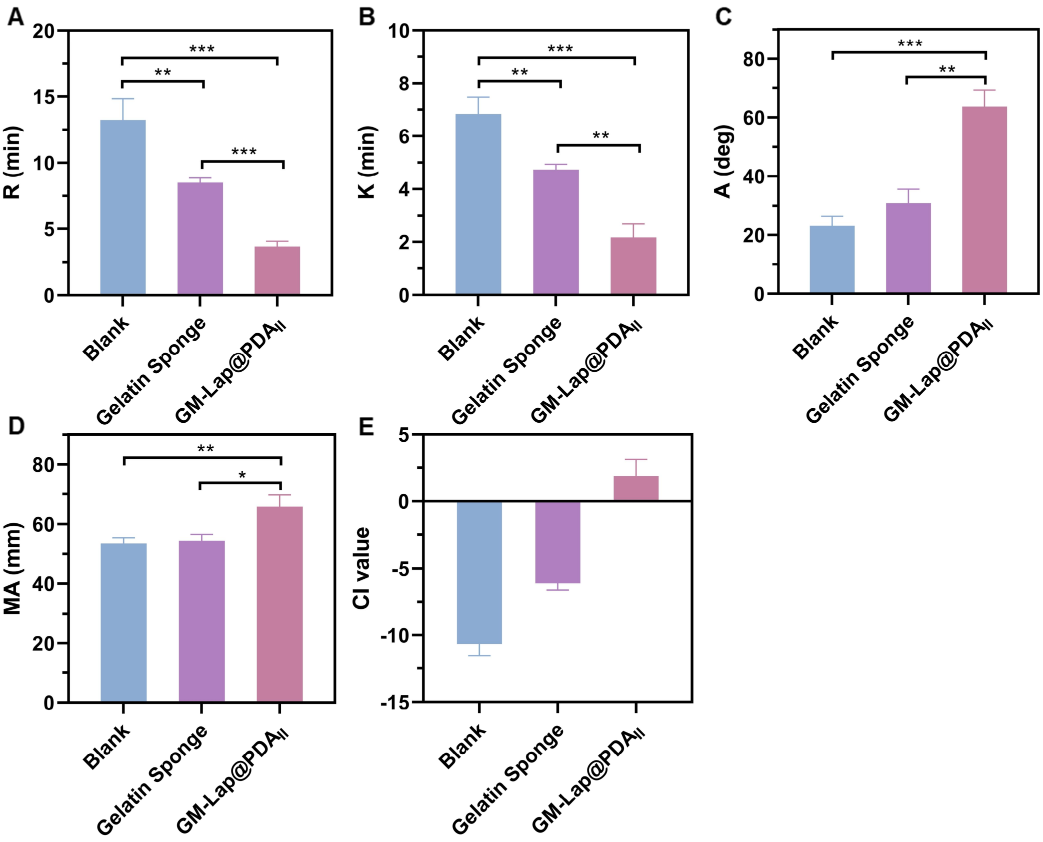

Figure S9. (A-E) TEG parameters of blank group, gelatin sponge and GM-Lap@PDAII hydrogel (n = 3). *$p < 0.05$, **$p < 0.01$, and ***$p < 0.001$.

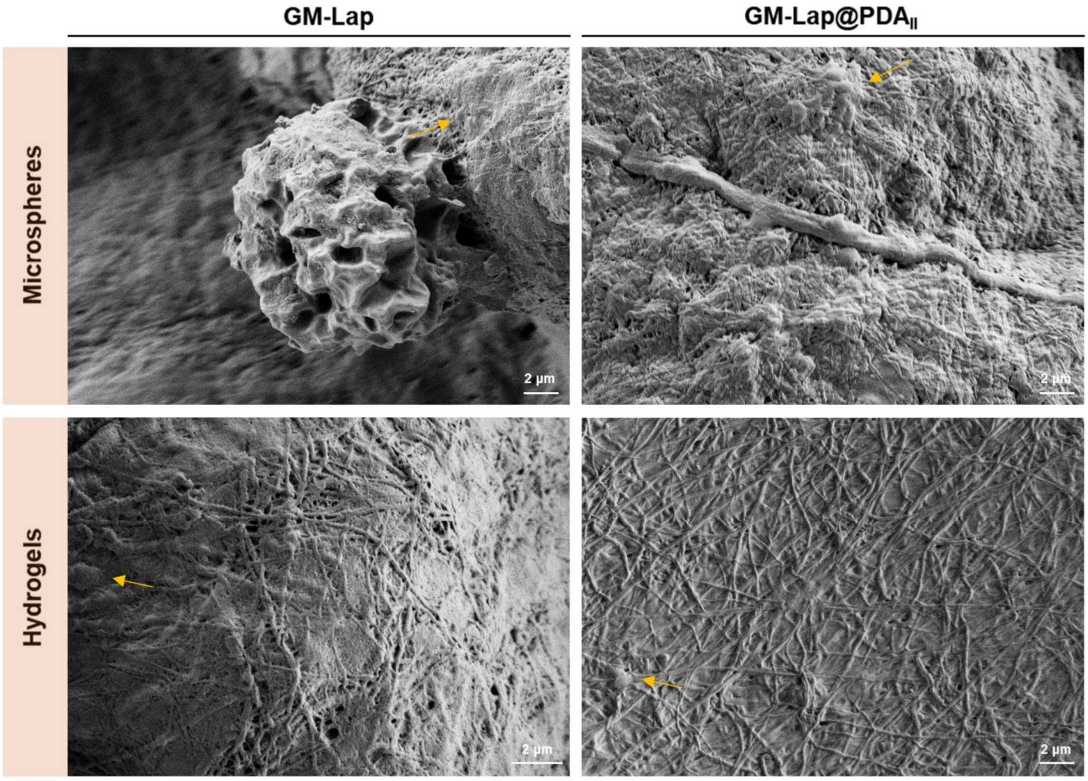

Figure S10. SEM images of platelet adhesion on the surface of GM-Lap@PDA cryogel microspheres and hydrogels.

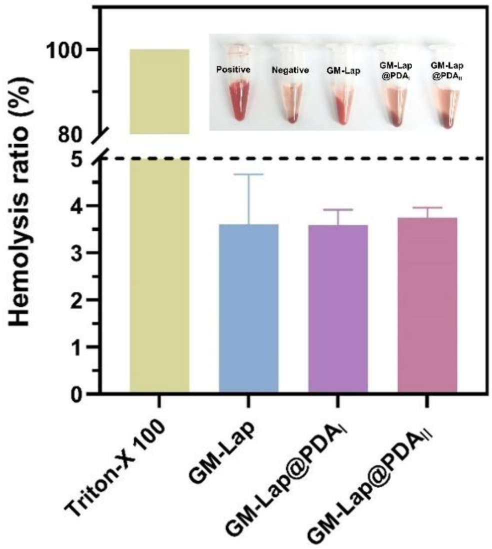

Figure S11. The hemolysis rates of GM-Lap@PDA hydrogels (n = 3).

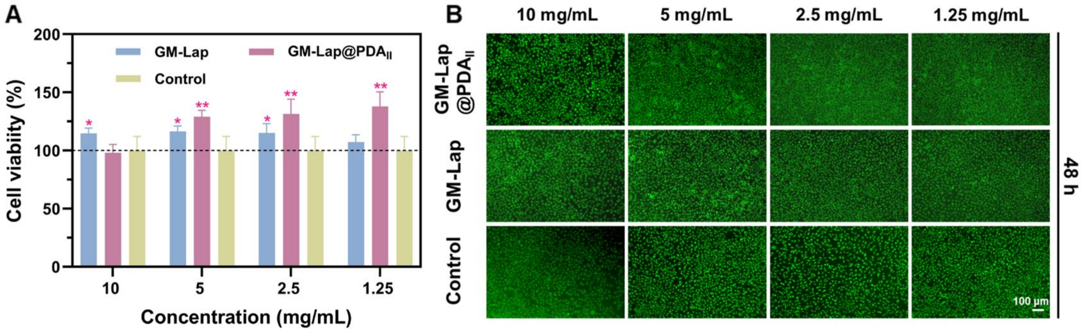

Figure S12. (A) Relative cell viability of L929 fibroblasts co-cultured with GM-Lap@PDA microspheres extracts for 48 h (the red asterisks represent the comparison of the experimental group with the control group at the same concentration, n = 5, **p* < 0.05 and ***p* < 0.01). (B) Live/dead cell fluorescence images (n = 4).

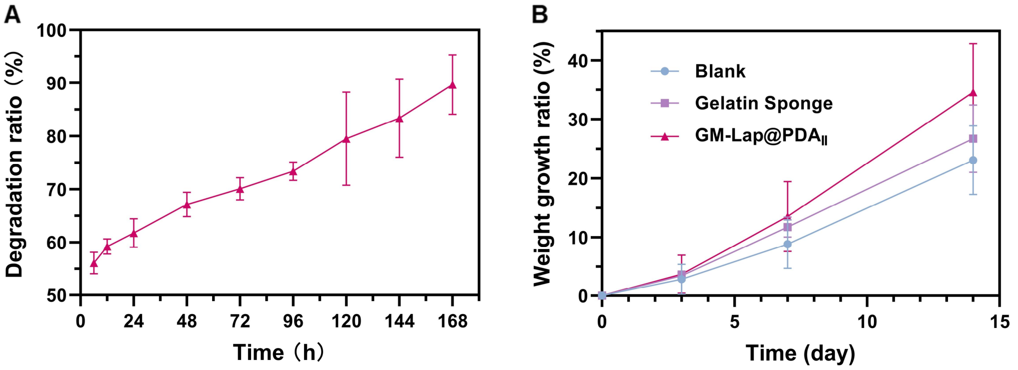

Figure S13. (A) *In vitro* enzymatic degradation profile of GM-Lap@PDA$_{II}$ hydrogel (n = 4). (B) The growth rates of body weight in rats at 3, 7 and 14 days after implantation surgery (n = 6).

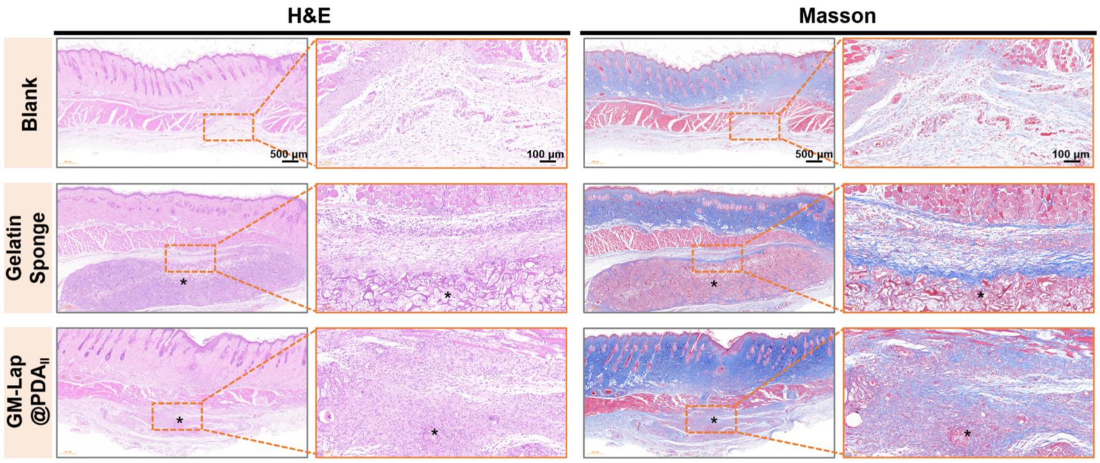

Figure S14. Representative images of H&E staining and Masson staining of tissue in the subcutaneous implantation area at 7 days postoperatively (* indicates the degraded residual material).

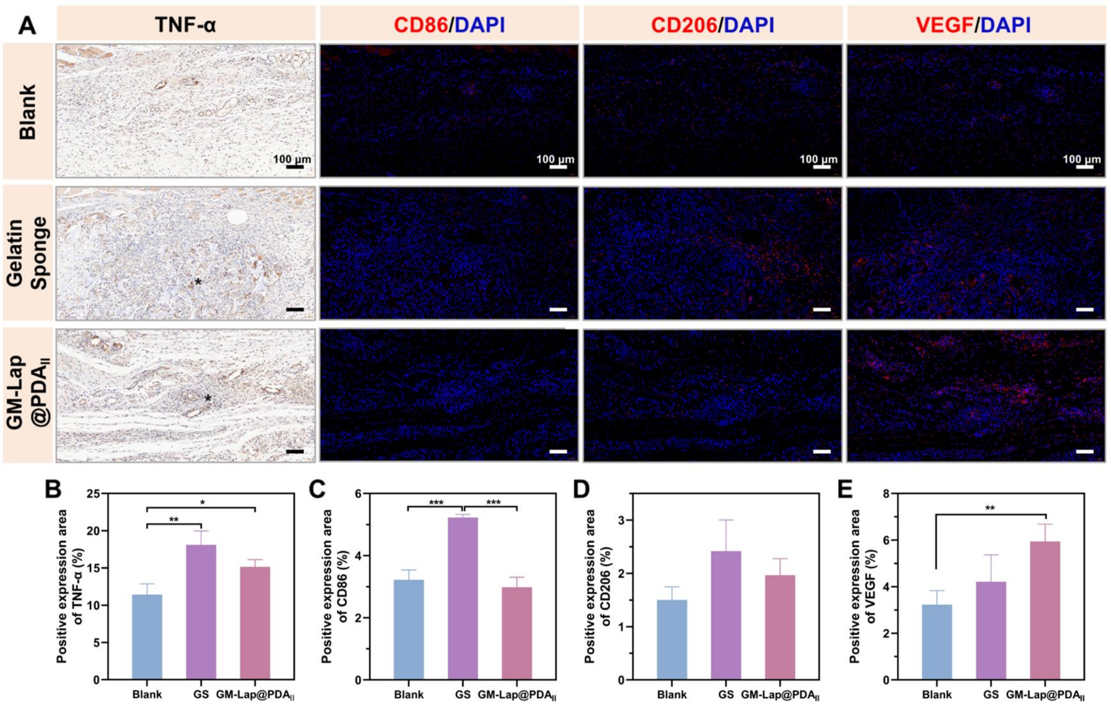

Figure S15. (A) Representative immunohistochemical staining and immunofluorescence images of tissue in the subcutaneous implantation area at 7 days postoperatively (* indicates the degraded residual material). (B-E) Quantitative analysis of TNF-α, CD86, CD206 and VEGF (n = 3). *$p < 0.05$, **$p < 0.01$, and ***$p < 0.001$.

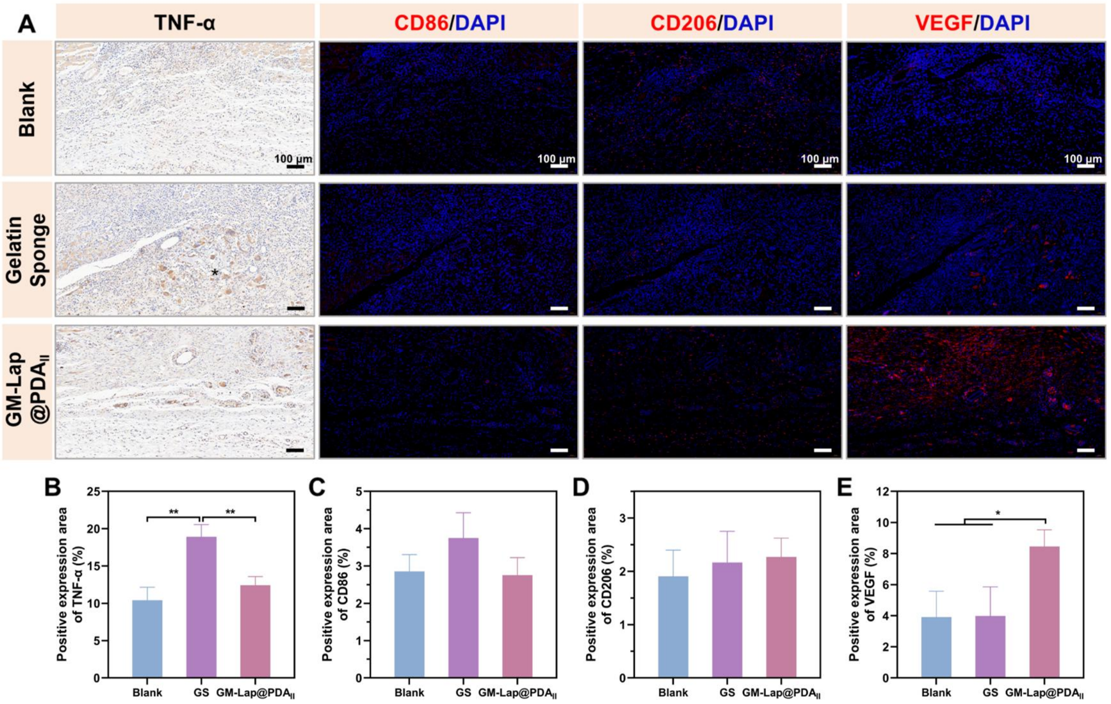

Figure S16. (A) Representative immunohistochemical staining and immunofluorescence images of tissue in the subcutaneous implantation area at 14 days postoperatively (* indicates the degraded residual material). (B-E) Quantitative analysis of TNF-α, CD86, CD206 and VEGF (n = 3). *$p < 0.05$ and **$p < 0.01$.

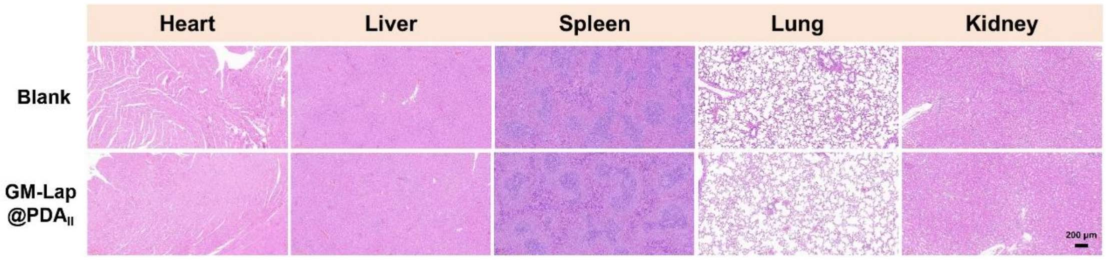

Figure S17. H&E staining images of major internal organs at 14 days postoperatively.

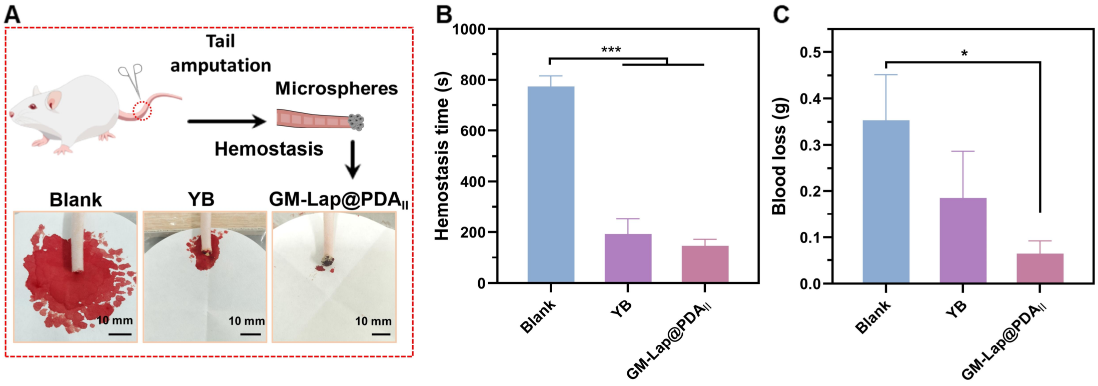

Figure S18. Hemostasis on rat tail amputation: (A) Schematic diagram of the rat tail amputation model and representative images of each group (the blank group, YB powder and GM-Lap@PDA$_{II}$ microspheres) at the completion of hemostasis. (B) Hemostasis time of each group. (C) Blood loss of each group (n = 4). *$p < 0.05$ and ***$p < 0.001$.

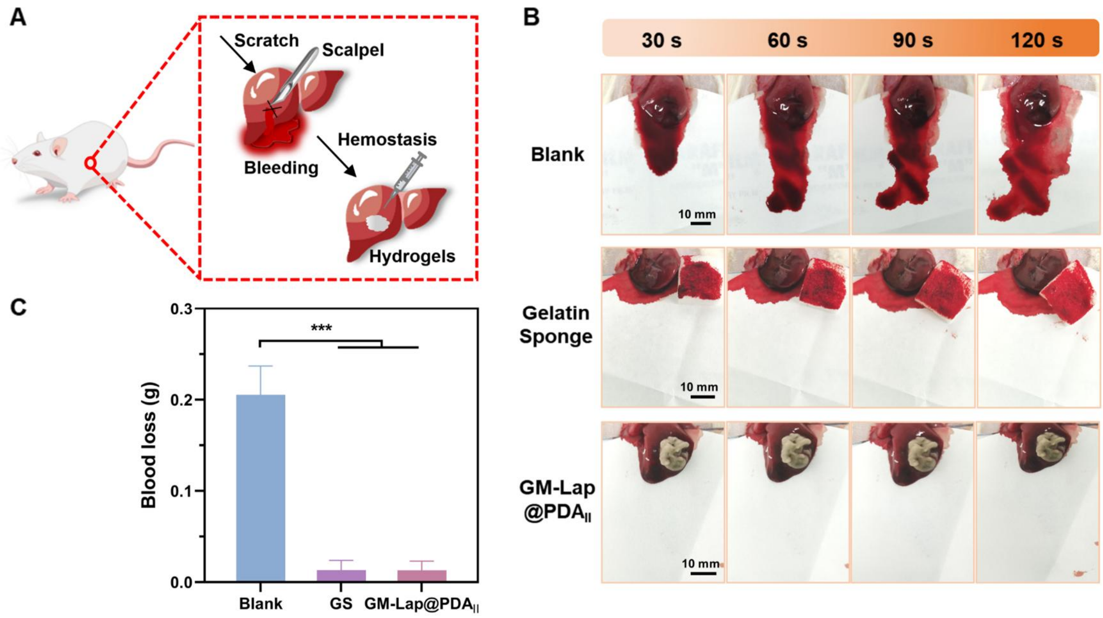

Figure S19. Hemostasis on rat liver cruciate incision: (A) Schematic diagram of the rat liver cruciate incision. (B) Representative images of the hemostatic process in each

group within 2 min. (C) Cumulative blood loss within 2 min in each group (the blank group, gelatin sponge (GS) and GM-Lap@PDA$_{II}$ hydrogel) (n = 4). ***$p < 0.001$.

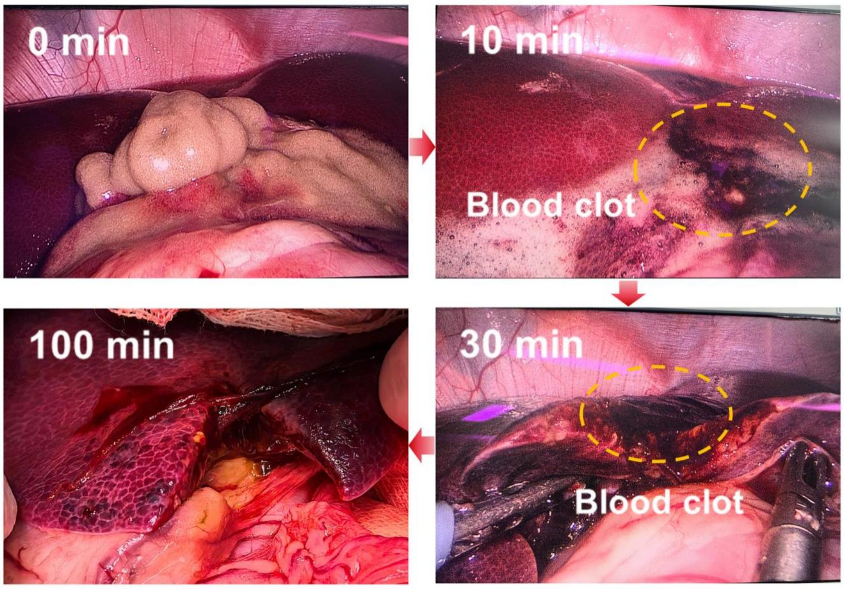

Figure S20. Hemostatic process after application of injectable hydrogel (GM-Lap@PDA$_{II}$) to the liver rupture wound under laparoscopic visualization. Following injection into the abdominal cavity and subsequent contact with blood, GM-Lap@PDA$_{II}$ hydrogel interacted with the blood components to form a large, tough clot that adhered to the wound surface.

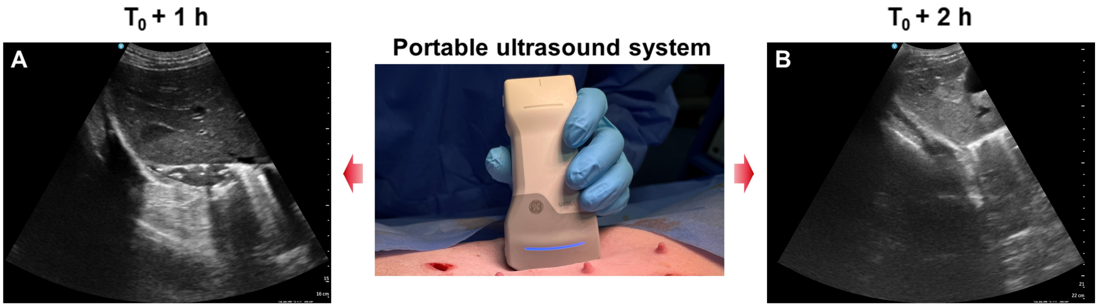

Figure S21. Examination of the injured area for further active hemorrhage using a portable ultrasound system: (A) Ultrasound image 1 hour after injury creation (T_0 + 1 h). (B) Ultrasound image 2 hour after injury creation (T_0 + 2 h).

Movie S1 Determination of water absorption by GM-Lap@PDA microspheres in 1s using optical video contact angle meter

Movie S2 Determination of blood absorption by GM-Lap@PDA microspheres in 1s using optical video contact angle meter

Movie S3 Hydration preparation and injection process of GM-Lap@PDA microsphere-based gels

Movie S4 Hemostasis on rat liver cruciate incision

Movie S5 Hemostasis on rat liver volumetric defect

Movie S6 Hemostasis on rat liver partial resection

Movie S7 Hemostasis on rabbit liver cruciate incision

Movie S8 Ultrasound-guided injection of hemostatic gel into an invisible bleeding site

Reference

[1] Huang Y, Fan C, Liu Y, Yang L, Hu W, Liu S, et al. Nature-derived okra gel as strong hemostatic bioadhesive in human blood, liver, and heart trauma of rabbits and dogs. Adv Healthcare Mater. 2022; 11: 2200939.

[2] Guo H, Zhang W, Jia Z, Wang P, Shao Q, Shen H, et al. A biodegradable supramolecular adhesive with robust instant wet adhesion for urgent hemostasis and wound repair. Adv Funct Mater. 2024; 34: 2401529.

[3] Wu K, Fu M, Zhao Y, Gerhard E, Li Y, Yang J, et al. Anti-oxidant anti-inflammatory and antibacterial tannin-crosslinked citrate-based mussel-inspired bioadhesives facilitate scarless wound healing. Bioact Mater. 2023; 20: 93.

[4] Yang Y, He G, Pan Z, Zhang K, Xian Y, Zhu Z, et al. An injectable hydrogel with ultrahigh burst pressure and innate antibacterial activity for emergency hemostasis and wound repair. Adv Mater. 2024; 36: 2404811.

[5] Zhang K, Xian Y, Li M, Pan Z, Zhu Z, Yang Y, et al. Gelable and adhesive powder for lethal non-compressible hemorrhage control. Adv Funct Mater. 2023; 33: 2305222.

[6] Lee M, Seo D, Park J, Lee SH, Jeon J, Kim W, et al. Wet tissue adhesive polymeric powder hydrogels for skeletal muscle regeneration. Bioact Mater. 2024; 40: 334.

[7] Liang M, Wei D, Yao Z, Ren P, Dai J, Xu L, et al. Hydrogel adhesive formed via multiple chemical interactions: from persistent wet adhesion to rapid hemostasis. Biomater Sci. 2022; 10: 1486.
